# Rapamycin preserves cardiac function in autoimmune myocarditis by reprogramming Cxcl9^+^ macrophages via the mTORC1–C/EBPβ–OSM axis

**DOI:** 10.1016/j.redox.2025.103970

**Published:** 2025-12-13

**Authors:** Yan Zhuang, Yongcui Yan, Zheng Wen, Xiaoquan Rao, Jiangang Jiang, Huihui Li, Dao Wen Wang

**Affiliations:** Division of Cardiology and Hubei Key Laboratory of Genetics and Molecular Mechanisms of Cardiological Disorders, Tongji Hospital, Tongji Medical College, Huazhong University of Science and Technology, Wuhan, China

**Keywords:** Myocarditis, Rapamycin, mTOR, Cxcl9^+^ macrophages, Inflammatory cardiomyopathy

## Abstract

**Background:**

Myocarditis is an inflammatory disease of the myocardium that can progress to chronic inflammatory cardiomyopathy and heart failure. Aberrant activation and metabolic reprogramming of macrophages drive myocardial inflammation and injury, yet effective targeted therapies remain limited.

**Methods:**

Experimental autoimmune myocarditis (EAM) was induced in BALB/c mice by α-myosin heavy chain immunization. Rapamycin was administered during the inflammatory phase. Cardiac function and injury were evaluated by echocardiography, Millar catheterization, histology, qPCR, and ELISA. Single-cell RNA sequencing (scRNA-seq) of cardiac CD45^+^ cells, coupled with pseudotime trajectory, SCENIC regulon, and NicheNet analyses, was performed to delineate macrophage heterogeneity, lineage dynamics, and macrophage–cardiomyocyte communication. Functional validation included Seahorse metabolic assays and Cebpb-overexpressing bone marrow–derived macrophage (BMDM)–cardiomyocyte co-culture experiments, along with in vivo OSM-neutralizing antibody (OSM-nAb) intervention.

**Results:**

Rapamycin preserved cardiac function and alleviated myocardial inflammation and fibrosis in EAM mice, accompanied by reduced cytokine release and cardiac injury markers. scRNA-seq revealed that rapamycin reprogrammed cardiac monocyte–macrophages by inhibiting mTOR signaling, restoring mitochondrial metabolism, and suppressing inflammatory, glycolytic, and senescence pathways. It specifically targeted pathogenic Cxcl9^+^ macrophages by disrupting the mTORC1–C/EBPβ axis and limiting their differentiation from Plac8^+^ monocytes. Rapamycin further protected cardiomyocytes by blocking C/EBPβ-dependent OSM-mediated macrophage–cardiomyocyte crosstalk. Therapeutic OSM neutralization in vivo similarly mitigated myocardial inflammation and fibrosis while preserving ventricular contractility.

**Conclusion:**

Rapamycin preserves cardiac function in autoimmune myocarditis by reprogramming Cxcl9^+^ macrophages via the mTORC1–C/EBPβ–OSM axis. Targeting OSM provides mechanistic validation and highlights a translational therapeutic strategy for myocarditis and chronic inflammatory cardiomyopathy.

## Graphical abstract

**Figure:**
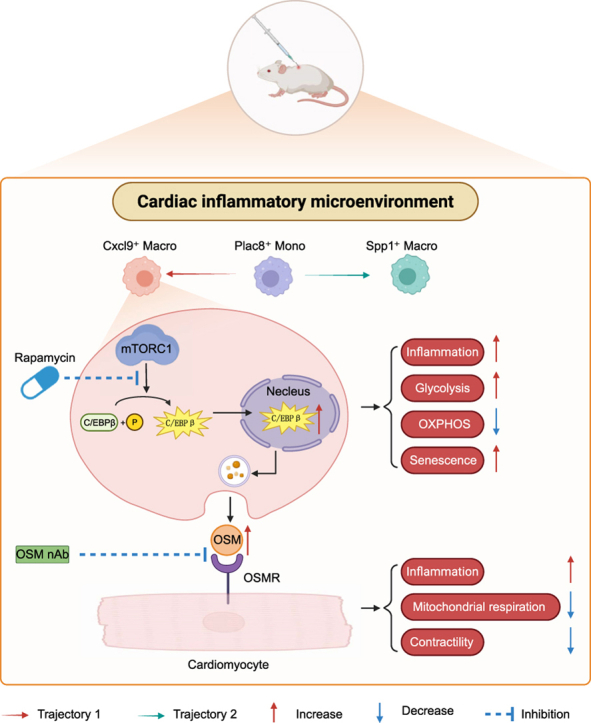

## 1 Introduction

Myocarditis is an inflammatory injury of the myocardium that can evolve into chronic inflammatory cardiomyopathy or dilated cardiomyopathy (DCM), constituting an important cause of heart failure worldwide [1,2]. Approximately 30 % of clinically overt myocarditis progresses to DCM over time, driving long-term morbidity and mortality [3]. Autopsy and biopsy series in DCM frequently reveal persistent inflammatory infiltrates and dysregulated immune signatures, supporting chronic inflammatory cardiomyopathy as a distinct phenotype [4,5]. Advances in hemodynamic support, guideline-directed heart failure management, and immunomodulatory strategies have substantially improved short-term survival during hospitalization [6,7]. Nevertheless, many survivors exhibit residual left-ventricular dysfunction, low-grade inflammation, and adverse remodeling during follow-up [8,9]. Even after apparent clinical recovery, a subset continues to show impaired contractility and biomarker elevation, underscoring an unmet need in the chronic inflammatory phase [[9], [10], [11]]. The mechanisms driving the transition from acute myocarditis to persistent inflammatory cardiomyopathy remain incompletely defined, and targeted therapies to interrupt this trajectory are lacking.

Emerging evidence indicates that immune activation, metabolic rewiring, and cellular senescence jointly sustain chronic cardiovascular inflammation [12]. The mechanistic target of rapamycin (mTOR) is a central serine/threonine kinase that assembles into at least two functionally distinct complexes: mTOR complex 1 (mTORC1, defined by Raptor) and mTOR complex 2 (mTORC2, defined by Rictor) [13,14]. mTORC1 serves as a rapamycin-sensitive hub integrating nutrient sensing with cell growth, bioenergetics, and stress responses, whereas mTORC2 is more prominently implicated in cytoskeletal organization and Akt-dependent survival signaling. In innate immune cells, mTORC1 regulates macrophage polarization, cytokine production, and glycolytic flux, coupling metabolic state to effector function [15,16]. Dysregulated cardiac mTOR signaling links metabolic dysfunction to maladaptive inflammation across atherosclerosis, myocardial infarction, and heart failure [17,18]. Pharmacological mTORC1 inhibition with rapamycin confers cardioprotective effects in preclinical settings—attenuating adverse remodeling, improving function, and modulating fibrotic and inflammatory responses [19]. Whether selective targeting of mTORC1 within cardiac immune compartments can disrupt pathogenic immune–cardiomyocyte crosstalk and forestall progression to chronic inflammatory cardiomyopathy, however, remains uncertain.

Within the IL-6 family, oncostatin M (OSM) has emerged as a gp130-utilizing cytokine produced predominantly by activated macrophages and T cells, with broad actions on stromal and parenchymal cells in chronic inflammatory states [20]. In the heart, OSM–OSMR/gp130 signaling has been identified as a potent modulator of cardiomyocyte phenotype and ventricular remodeling. Experimental studies show that OSM can induce cardiomyocyte dedifferentiation and structural remodeling during acute myocardial infarction and chronic DCM, with context-dependent effects on function and outcome [21]. Continuous activation of the OSM receptor complex can drive progressive heart failure, whereas carefully titrated or transient signaling may support cardiomyocyte protection and regeneration, underscoring its dual roles in injury versus chronic remodeling [22]. These observations implicate OSM–OSMR pathways as attractive but pharmacologically challenging targets in inflammatory cardiomyopathies.

In this study, we interrogated the role of mTORC1-mediated immune–metabolic regulation in chronic inflammatory cardiomyopathy by combining experimental autoimmune myocarditis (EAM), single-cell transcriptomics, metabolic analysis, and cardiomyocyte functional assays. We specifically test whether rapamycin modulates cardiac monocyte–macrophage states and their signaling to cardiomyocytes, with a focus on a C/EBPβ-centered transcriptional network and OSM-mediated intercellular communication. Neutralization of OSM in vivo recapitulates key protective effects, supporting the mTORC1–C/EBPβ–OSM axis as a plausible translational target in myocarditis and chronic inflammatory cardiomyopathy.

## 2 Methods

### 2.1 Data availability

All detailed experimental procedures [animal models, cardiomyocyte isolation, cell culture, high-throughput sequencing, immunoblotting, Seahorse assays, immunostaining, and quantitative PCR (qPCR)], along with reagent lists (primers, antibodies) are provided in the Supplemental Material.

### 2.2 Ethics approvals

All animal procedures conformed to the Guide for the Care and Use of Laboratory Animals (U.S. National Research Council) and were approved by the Institutional Animal Care and Use Committee of Tongji Medical College, Huazhong University of Science and Technology (approval No. 4364). Efforts were made to minimize animal suffering, reduce animal numbers, and use alternatives whenever possible. Investigators were blinded to treatment allocation during echocardiographic/hemodynamic acquisition and histological quantification.

### 2.3 Statistical analysis

Statistical analyses were performed using GraphPad Prism (v10) and R (v4.3). Normality was assessed with the Shapiro–Wilk test. Continuous data are presented as mean ± SEM (normally distributed) or median [IQR] (non-normal). Two-group comparisons used two-tailed Student's *t*-test (parametric) or Mann–Whitney *U* test (non-parametric). Comparisons among ≥3 groups used one-way or two-way ANOVA with Tukey's or Bonferroni post-hoc tests, as appropriate. Categorical variables were compared with χ^2^ or Fisher's exact tests. Correlations used Spearman's ρ. For Seahorse assays and repeated measurements, between-group effects were evaluated by two-way ANOVA with interaction terms and appropriate multiple-comparison correction. For single-cell analyses, quality control, normalization, dimensionality reduction, clustering, and differential expression followed standard pipelines, with gene-level statistics obtained by Wilcoxon rank-sum tests and Benjamini–Hochberg false-discovery rate (FDR) correction. Gene-set scoring used AddModuleScore/AUCell, and pathway enrichment employed clusterProfiler/fgsea with FDR control. Pseudotime trajectories were inferred with Monocle 3; regulon activity was computed via SCENIC/AUCell; ligand–receptor inference used NicheNet. Subset enrichment/odds ratios were estimated from cell-type proportions using logistic models with cluster-robust standard errors; 95 % confidence intervals are reported where applicable. Unless otherwise stated, tests were two-sided, and *P* < 0.05 (or FDR q < 0.05 for multi-gene analyses) was considered statistically significant.

## 3 Results

### 3.1 Rapamycin preserves cardiac function and mitigates inflammation and fibrosis in EAM

**Fig. 1:**
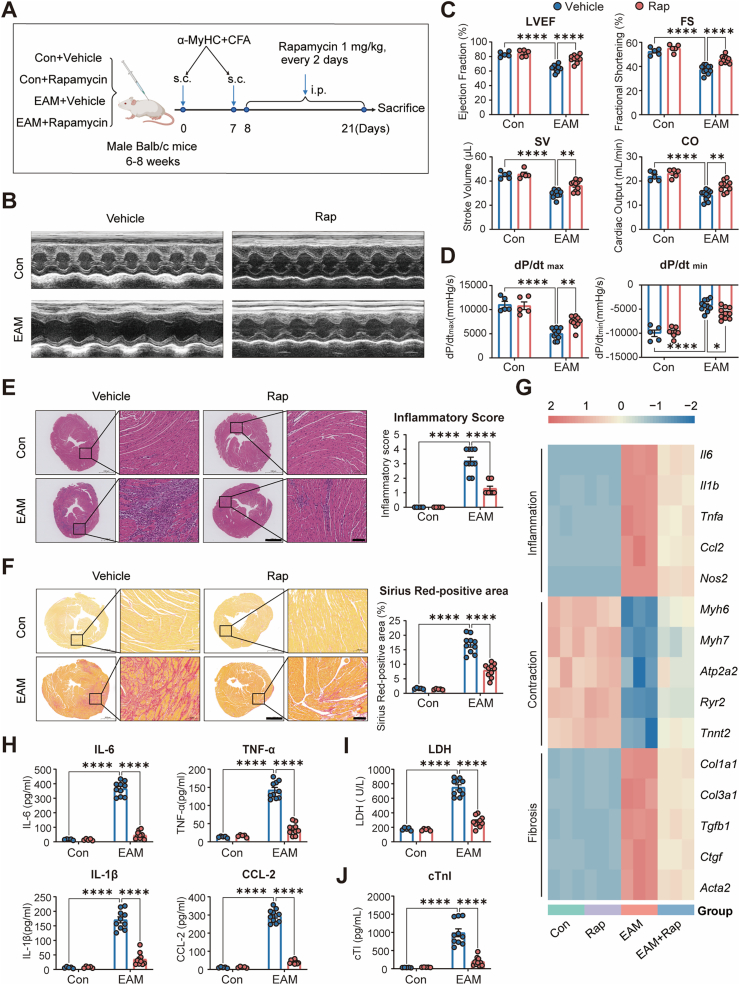
**Rapamycin preserves cardiac function and mitigates myocardial inflammation and fibrosis in EAM.** **A**, Schematic of the experimental design. Male BALB/c mice received subcutaneous (s.c.) injections of α-myosin heavy chain (α-MyHC) emulsified in complete Freund's adjuvant (CFA) on days 0 and 7 to induce experimental autoimmune myocarditis (EAM). Rapamycin (1 mg kg^−1^, intraperitoneally [i.p.], every 2 days) or vehicle was administered from day 8 to day 21. Four groups were studied: Control + Vehicle, Control + Rapamycin, EAM + Vehicle, and EAM + Rapamycin. **B**, Representative M-mode echocardiographic images of each group. **C**, Quantification of EF, FS, SV, and CO (n = 5, 5, 10, and 10 for Control + Vehicle, Control + Rapamycin, EAM + Vehicle, and EAM + Rapamycin, respectively). **D**, Hemodynamic assessment of left-ventricular contractility showing peak rates of pressure increase (dP/dtmax) and decline (dP/dtmin) measured by Millar catheterization (n = 5, 5, 10, and 10 per group as in C). **E**, Representative H&E staining and quantitative analysis of heart sections illustrating inflammatory cell infiltration (n = 5, 5, 10, and 10 per group); Scale bars: 1 mm in low-magnification overview images and 100 μm in high-magnification inset images. **F**, Sirius Red staining and quantitative analysis showing interstitial collagen deposition (n = 5, 5, 10, and 10 per group); Scale bars: 1 mm in low-magnification overview images and 100 μm in high-magnification inset images. **G**, Heatmap of cardiac mRNA expression by RT-qPCR for representative genes grouped by inflammation (e.g., *Il6, Tnfa, Il1b, Ccl2, Nos2*), contraction (e.g., *Atp2a2, Ryr2, Tnnt2, Myh6*), and fibrosis (e.g., *Col1a1, Col3a1, Tgfb1, Ctgf, Acta2*) (n = 3 per group). **H**, Plasma concentrations of IL-6, TNF-α, IL-1β, and CCL2 determined by ELISA (n = 5, 5, 10, and 10 per group). **I** and **J**, Plasma LDH and cardiac troponin I levels as indicators of myocardial injury (n = 5, 5, 10, and 10 per group). Data are presented as mean ± SEM. ∗*P* < 0.05, ∗∗*P* < 0.01, ∗∗∗*P* < 0.001, ∗∗∗∗*P* < 0.0001 by one-way or two-way ANOVA followed by Tukey's multiple-comparison test. **Abbreviations:** EAM, experimental autoimmune myocarditis; α-MyHC, α-myosin heavy chain; CFA, complete Freund's adjuvant; s.c., subcutaneous; i.p., intraperitoneal; EF, ejection fraction; FS, fractional shortening; SV, stroke volume; CO, cardiac output; dP/dtmax, peak rate of LV pressure rise; dP/dtmin, peak rate of LV pressure decline; H&E, hematoxylin and eosin; RT-qPCR, reverse transcription quantitative PCR; ELISA, enzyme-linked immunosorbent assay; LDH, lactate dehydrogenase; SEM, standard error of the mean; ANOVA, analysis of variance.

To evaluate the therapeutic potential of rapamycin in chronic inflammatory cardiomyopathy, we established an EAM model in BALB/c mice through cardiac myosin immunization, followed by rapamycin intervention initiated during the inflammatory phase (Fig. 1A). Echocardiographic assessment revealed that EAM mice exhibited marked cardiac dysfunction, characterized by reductions in left ventricular ejection fraction (LVEF), fractional shortening (FS), stroke volume (SV), and cardiac output (CO). Notably, rapamycin treatment significantly preserved cardiac function, as evidenced by improvements in LVEF, FS, SV, and CO compared to vehicle-treated EAM mice (Fig. 1B and C). Consistently, hemodynamic assessment by Millar catheterization demonstrated that rapamycin markedly improved ventricular contractile and relaxation performance, as indicated by increased dP/dt_max_ and reduced dP/dt_min_ (Fig. 1D).

Histological analysis revealed pronounced myocardial inflammation and interstitial fibrosis in EAM hearts, both of which were markedly attenuated by rapamycin. H&E staining demonstrated reduced inflammatory cell infiltration (Fig. 1E), and Sirius Red staining confirmed a significant decrease in collagen deposition (Fig. 1F). At the molecular level, rapamycin suppressed the transcriptional induction of inflammatory cytokines (*Il6, Tnfa*) and fibrosis markers genes (*Col1a1, Tgfb1*) while sustaining the expression of cardiomyocyte contractile and mitochondria genes (*Atp2a2, Ryr2*) in EAM hearts (Fig. 1G). Consistently, ELISA revealed elevated plasma IL-6, TNF-α, IL-1β, and CCL2 levels in EAM mice, which were significantly reduced by rapamycin (Fig. 1H). Furthermore, cardiac injury biomarkers, including plasma lactate dehydrogenase (LDH) and cardiac troponin I (cTnI), were significantly elevated in EAM mice and attenuated by rapamycin treatment (Fig. 1I and J).

Collectively, these findings demonstrate that rapamycin mitigates myocardial inflammation, fibrosis, and injury, while preserving cardiac function in EAM.

### 3.2 Rapamycin reprograms cardiac Monocyte–Macrophage metabolic and inflammatory states in EAM

**Fig. 2:**
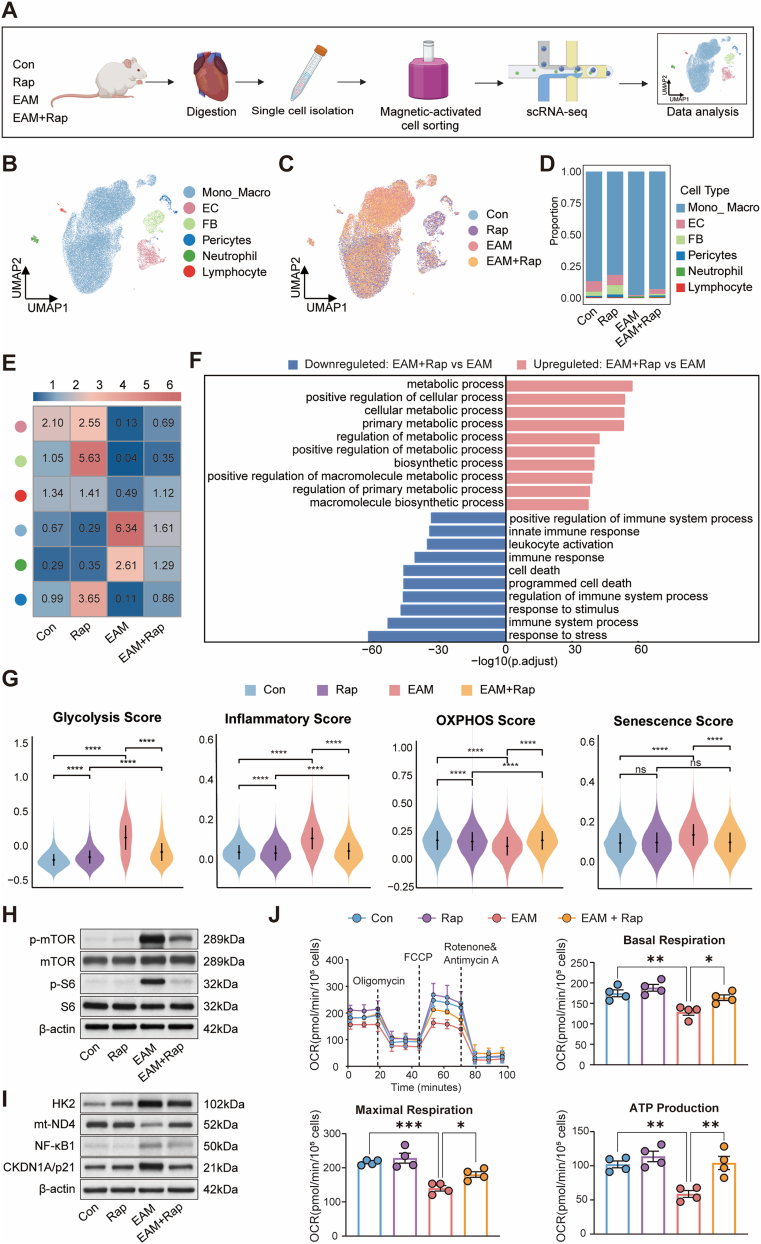
**Rapamycin reprograms cardiac monocyte–macrophage states in EAM.** **A**, Workflow for scRNA-seq of cardiac CD45^+^ immune cells from Control (Con), Rapamycin (Rap), EAM, and EAM + Rap groups, including tissue digestion, single-cell isolation, magnetic-activated cell sorting, library preparation, and data analysis. **B**, UMAP visualization of major cardiac immune compartments identified by unsupervised clustering and annotation. **C**, UMAP plots showing distribution of cells from each experimental group. **D**, Stacked bar charts showing the proportional composition of immune/stromal subsets among groups. **E**, Heatmap of odds ratios depicting enrichment of each subset across groups. **F**, GO enrichment of differentially expressed genes in the monocyte–macrophages (Mono_Macro) compartment comparing EAM and EAM + Rap. **G**, Gene set scoring of glycolysis, oxidative phosphorylation, inflammatory response, and senescence signatures in the myeloid compartment across groups. **H**–**I**, immunoblotting analysis of isolated cardiac macrophages showing phosphorylation of mTOR and S6 (**H**), and levels of HK2, NF-κB1, CDKN1A/p21, and mitochondrial complex I subunit mt-ND4 (**I**); β-actin serves as loading control. **J**, Seahorse analysis of OCR in isolated cardiac macrophages, with representative traces and quantification of basal respiration, maximal respiration, and ATP production (n = 4/group). Data are presented as mean ± SEM. ns indicates no significant difference, ∗*P* < 0.05, ∗∗*P* < 0.01, ∗∗∗*P* < 0.001, ∗∗∗∗*P* < 0.0001 by one-way or two-way ANOVA followed by Tukey's multiple comparisons test. **Abbreviations:** EAM, experimental autoimmune myocarditis; Rap, rapamycin; scRNA-seq, single-cell RNA sequencing; UMAP, uniform manifold approximation and projection; Mono_Macro, monocyte–macrophage; OR, odds ratio; GO, Gene Ontology; OCR, oxygen consumption rate; FCCP, carbonyl cyanide-p-trifluoromethoxyphenylhydrazone; SEM, standard error of the mean; ANOVA, analysis of variance.

To delineate the immunoregulatory landscape modulated by rapamycin in EAM, single-cell RNA sequencing (scRNA-seq) was performed on cardiac CD45^+^ immune cells isolated from control (Con), rapamycin-treated (Rap), EAM, and EAM + Rap mice (Fig. 2A). Unsupervised UMAP projection coupled with canonical marker annotation identified five principal immune and stromal lineages—monocyte–macrophages (Mono_Macro), neutrophils, lymphocytes, endothelial cells (ECs), and fibroblasts (FBs)—along with a minor pericyte subset (Fig. 2B; Fig. S1A–C). Comparative analysis across conditions revealed a marked expansion of the Mono_Macro population in EAM hearts, which was notably attenuated following rapamycin treatment (Fig. 2C and D). Odds ratio (OR) analysis further demonstrated that Mono_Macro represented the most significantly enriched cell type in EAM, exhibiting the highest OR among all lineages and indicating a pronounced disease-associated expansion consistent with previous observations [23]. Rapamycin markedly decreased the OR of Mono_Macro, reflecting its preferential suppressive effect on this population (Fig. 2E). Consistently, Mono_Macro exhibited the largest number of rapamycin-responsive genes among all lineages (Fig. S1D), underscoring its predominant transcriptional reprogramming under treatment. These findings identify monocyte–macrophages as the major effector population in EAM and the principal cellular target of rapamycin.

Gene Ontology (GO) enrichment analysis showed that rapamycin partially reversed the transcriptional alterations in EAM macrophages, suppressing immune-related programs (e.g., immune system process, leukocyte activation, innate immune response, response to stress) while restoring metabolic and biosynthetic pathways (Fig. 2F). Correspondingly, pathway activity scoring indicated that EAM Mono_Macro underwent a metabolic–inflammatory shift characterized by enhanced glycolysis and inflammatory signaling, reduced oxidative phosphorylation (OXPHOS), and increased cellular senescence—all of which were mitigated by rapamycin treatment (Fig. 2G). Gene Set Enrichment Analysis (GSEA) further confirmed these findings: senescence, glycolysis, and inflammatory signatures were positively enriched in EAM macrophages, whereas mitochondrial OXPHOS was negatively enriched; rapamycin effectively reversed these transcriptional patterns (Fig. S1E). Consistently, genes involved in inflammatory stress and glycolytic activation (*Cdkn1a, S100a6, Cxcl9, Nfkb1, Hk2, Aldoa*) were upregulated in EAM, while mitochondrial respiratory complex genes (*mt-Nd4, Ndufa12*) were downregulated; rapamycin treatment restored their expression (Fig. S1F).

To validate these transcriptional adaptations at the protein and functional level, cardiac macrophages were isolated via F4/80 magnetic sorting for immunoblotting and Seahorse metabolic assays. Western blot analysis confirmed that rapamycin effectively inhibited mTOR signaling, as shown by decreased phosphorylation of mTOR and S6, and concurrently downregulated glycolytic enzyme HK2, inflammatory mediator NF-κB1, and senescence marker p21, while restoring mitochondrial protein mt-ND4 (Fig. 2H and I). In parallel, pathway scoring analysis did not reveal a consistent activation of the mTORC2 pathway in cardiac macrophages across conditions, nor a major rapamycin-induced shift (Fig. S1G). Consistent with these molecular changes, Seahorse assays demonstrated that rapamycin significantly improved mitochondrial oxidative phosphorylation, reflected by enhanced basal and maximal respiration as well as ATP production in EAM macrophages (Fig. 2J).

Collectively, these findings demonstrate that rapamycin reprograms cardiac Mono_Macro in EAM by suppressing mTOR signaling, restoring mitochondrial metabolic competence, and attenuating inflammatory and senescence-associated activation.

### 3.3 Rapamycin targets pathogenic Cxcl9^+^ macrophages to reverse inflammatory and metabolic reprogramming in EAM

**Fig. 3:**
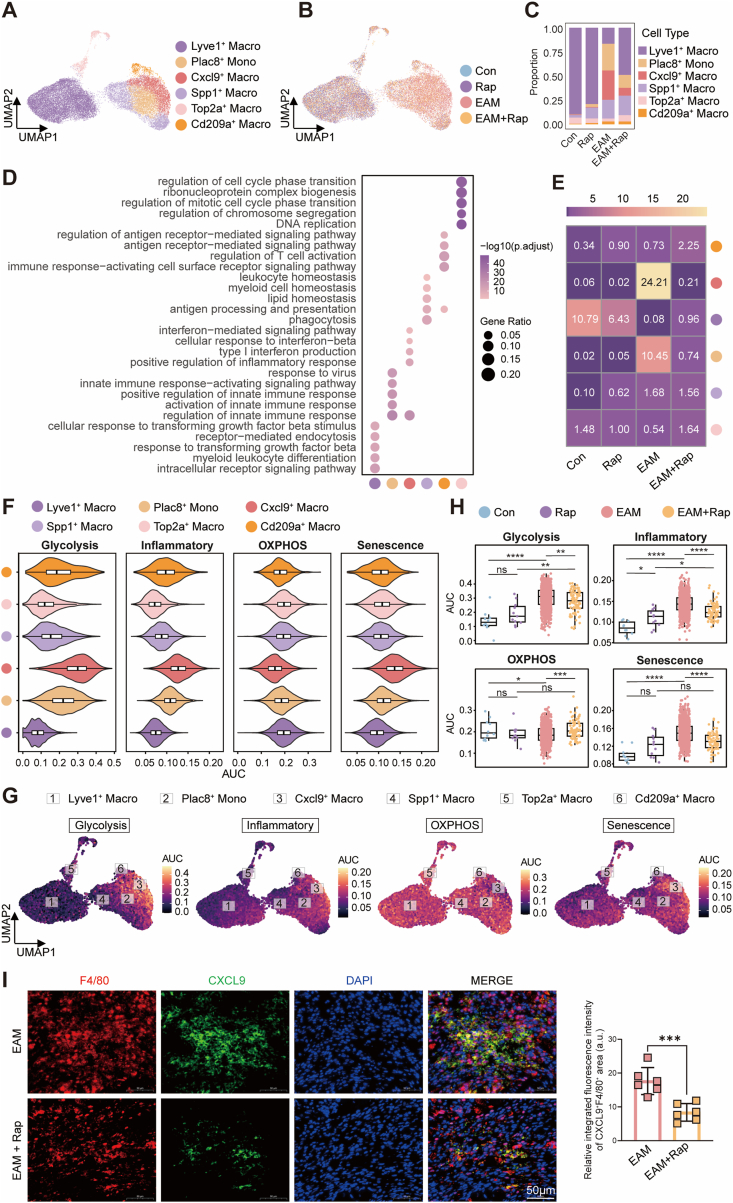
**Rapamycin targets a pathogenic Cxcl9^+^ macrophage subset and reshapes inflammatory–metabolic states in EAM.** **A**, UMAP visualization of reclustered monocyte–macrophage populations from cardiac single-cell RNA-seq, identifying Lyve1^+^, Plac8^+^, Cxcl9^+^, Spp1^+^, Top2a^+^, and Cd209a^+^ subsets. **B**, UMAP plots showing distribution of monocyte–macrophage subsets across experimental groups [Control (Con), Rapamycin (Rap), EAM, and EAM + Rap]. **C**, Proportional changes of monocyte–macrophage subsets among groups. **D**, GO enrichment analysis of biological processes associated with each macrophage subset. **E**, Heatmap showing odds ratios of enrichment of macrophage subsets across experimental groups. **F**, AUCell-based pathway scoring illustrating glycolysis, inflammatory, OXPHOS, and senescence signatures across macrophage subsets. **G**, UMAP feature plots displaying the spatial distribution and activity scores (AUC) of glycolysis, inflammatory, OXPHOS, and senescence pathways across macrophage subsets. **H**, Quantitative comparison of pathway activity scores in Cxcl9^+^ macrophages among Con, Rap, EAM, and EAM + Rap groups. **I**, Representative immunofluorescence staining of F4/80 and CXCL9 in cardiac tissue sections from EAM and EAM + Rapamycin groups, with quantification of CXCL9 signal intensity (n = 6 per group). Scale bar, 50 μm. Data are shown as mean ± SEM. ns indicates no significant difference, ∗*P* < 0.05, ∗∗*P* < 0.01, ∗∗∗*P* < 0.001, ∗∗∗∗*P* < 0.0001 by one-way or two-way ANOVA with Tukey's multiple-comparison test. **Abbreviations:** UMAP, uniform manifold approximation and projection; scRNA-seq, single-cell RNA sequencing; GO, Gene Ontology; OXPHOS, oxidative phosphorylation; AUC, area under the curve; EAM, experimental autoimmune myocarditis; Rap, rapamycin; CXCL9, C-X-C motif chemokine ligand 9; SEM, standard error of the mean; ANOVA, analysis of variance.

To resolve macrophage heterogeneity and identify rapamycin-responsive subpopulations, the Mono_Macro compartment from the cardiac scRNA-seq dataset was reclustered, revealing six transcriptionally distinct subsets: Lyve1^+^ tissue-resident macrophages, Plac8^+^ monocytes, Cxcl9^+^ inflammatory macrophages, Spp1^+^ remodeling macrophages, Top2a^+^ proliferative macrophages, and Cd209a^+^ antigen-presenting macrophages (Fig. 3A; Fig. S2A–B). Condition-specific UMAP distribution revealed a prominent expansion of Cxcl9^+^ macrophages in EAM, accompanied by an increase in Plac8^+^ monocytes and a marked depletion of Lyve1^+^ macrophages, all of which were at least partially normalized by rapamycin treatment (Fig. 3B and C). GO enrichment analysis highlighted distinct functional programs across subsets (Fig. 3D): Lyve1^+^ macrophages were associated with leukocyte and myeloid homeostasis, Plac8^+^ monocytes with early innate immune activation, Cxcl9^+^ macrophages with interferon-mediated inflammatory responses, Spp1^+^ macrophages with phagocytosis and matrix remodeling, Cd209a^+^ macrophages with antigen presentation, and Top2a^+^ macrophages with cell-cycle regulation. OR analysis further confirmed that, among these subsets, Cxcl9^+^ macrophages represented the most disease-enriched population (OR = 24.21) and exhibited the greatest sensitivity to rapamycin suppression (OR = 0.21) (Fig. 3E).

To further characterize the functional states of these subsets, AUCell-based pathway scoring revealed pronounced metabolic and inflammatory heterogeneity across clusters (Fig. 3F and G). Notably, Cxcl9^+^ macrophages displayed the highest glycolytic and inflammatory activity together with a strong senescence signature and a marked suppression of OXPHOS, indicative of a metabolically dysregulated inflammatory phenotype in EAM. Rapamycin treatment reprogrammed this subset, significantly attenuating glycolytic flux, inflammatory activation, and senescence, while partially restoring mitochondrial oxidative metabolism (Fig. 3H). Consistently, GO analysis and GSEA of Cxcl9^+^ macrophages showed that rapamycin downregulated inflammatory and stress-response pathways while reactivating metabolic and biosynthetic processes, including oxidative phosphorylation and macromolecule synthesis (Fig. S2C–E). At the gene level, glycolysis-related genes (*Hk2, Slc2a1*), inflammatory chemokines (*Cxcl9*, *Ccl5*), and senescence-associated markers (*Cdkn1a*, *S100a6*) were highly expressed in EAM and markedly attenuated by rapamycin. Conversely, mitochondrial respiratory chain components (*Uqcrc1*, *Park7*) were reduced in EAM but restored upon treatment (Fig. S2F). Immunofluorescence staining validated the expansion of F4/80^+^CXCL9^+^ macrophages in EAM hearts, and their accumulation was markedly diminished following rapamycin administration (Fig. 3I), consistent with the scRNA-seq findings.

Collectively, these findings identify Cxcl9^+^ macrophages as the major pathogenic subset in EAM, characterized by inflammatory, glycolytic, and senescence-driven metabolic reprogramming. Rapamycin targets this population, reversing mTOR-dependent inflammatory activation and restoring mitochondrial metabolic balance.

### 3.4 Rapamycin remodels the differentiation and metabolic reprogramming of pathogenic Cxcl9^+^ macrophages by suppressing the mTORC1–C/EBPβ axis

**Fig. 4:**
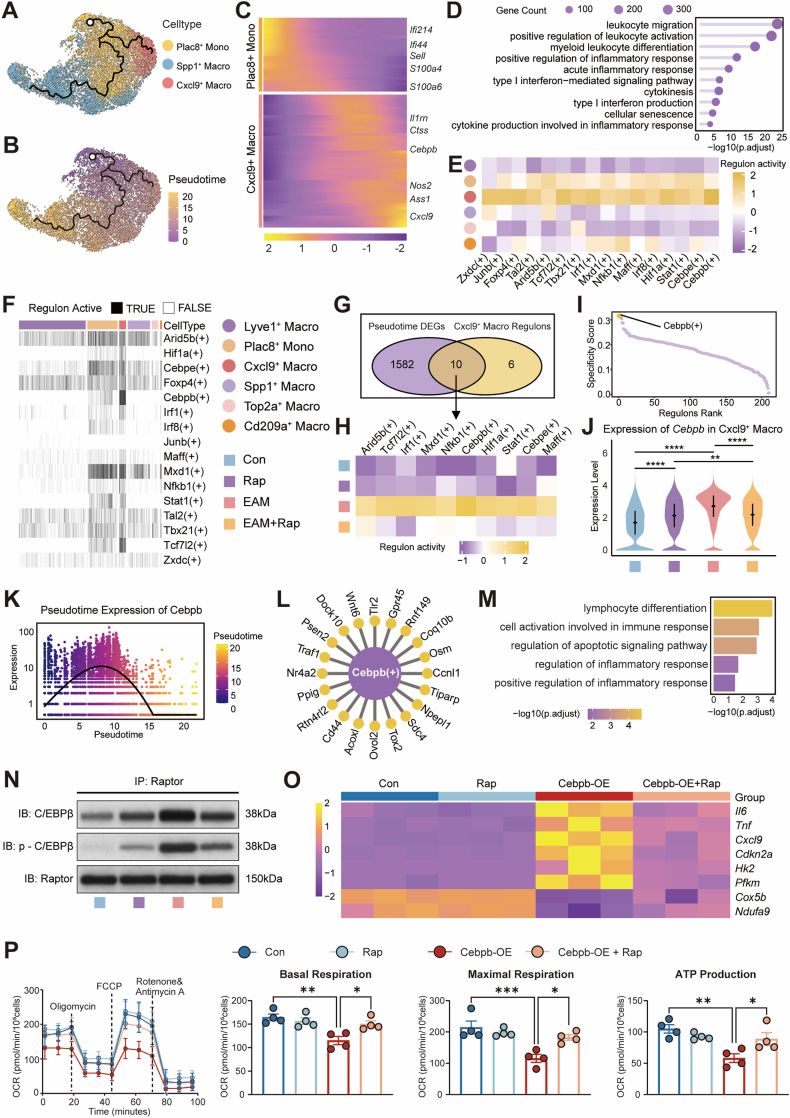
**Rapamycin reshapes the differentiation landscape and transcriptional control of pathogenic Cxcl9^+^ macrophages via the mTORC1–C/EBPβ axis.** **A**, UMAP visualization of reclustered cardiac monocyte–macrophages with subset annotation, including Plac8^+^ monocytes, Spp1^+^ macrophages, and Cxcl9^+^ macrophages; black line indicates the Monocle3-inferred trajectory and white circle denotes the root. **B**, UMAP plot of the same embedding colored by pseudotime. **C**, Heatmaps showing representative gene expression dynamics along pseudotime for the Plac8^+^ monocyte and Cxcl9^+^ macrophage branches. **D**, GO enrichment analysis of pseudotime-associated genes along the Plac8^+^ to Cxcl9^+^ branch. **E**, Heatmap depicting regulon activity scores of selected transcriptional regulators identified along macrophage differentiation trajectories. **F**, Binary heatmap showing SCENIC-inferred regulon activation across macrophage subsets, with cell type and experimental group annotations. **G**, Venn diagram illustrating the overlap between pseudotime-dependent DEGs and key regulons identified in Cxcl9^+^ macrophages. **H**, Heatmap displaying regulon activity of selected transcriptional regulators across experimental groups. **I**, Specificity ranking of the Cebpb regulon in Cxcl9^+^ macrophages based on AUCell-derived activity scores. **J**, Violin plots showing *Cebpb* expression levels in Cxcl9^+^ macrophages across experimental groups. **K**, Pseudotime plot depicting dynamic changes in *Cebpb* expression along the Plac8^+^ monocyte–to–Cxcl9^+^ macrophage differentiation trajectory. **L**, Network representation of the C/EBPβ regulon showing predicted downstream targets in Cxcl9^+^ macrophages. **M**, GO enrichment analysis of C/EBPβ target genes. **N**, Co-immunoprecipitation of Raptor followed by immunoblotting for total and phosphorylated C/EBPβ in cardiac macrophages from Con, Rap, EAM, and EAM + Rap groups. **O**, RT-qPCR analysis of inflammatory (*Il6, Tnf, Cxcl9*), glycolytic (*Hk2, Pfkm*), and senescence-associated (*Cdkn2a*) transcripts in BMDMs transduced with Ad-Cebpb or control and treated with vehicle or rapamycin (n = 4/group). RT-qPCR results are presented as a heatmap. **P**, Seahorse extracellular flux analysis of OCR in BMDMs from the same groups, including representative OCR trace and quantification of basal respiration, maximal respiration, and ATP production (n = 4/group). Data are shown as mean ± SEM unless indicated. ns indicates no significant difference, ∗*P* < 0.05, ∗∗*P* < 0.01, ∗∗∗*P* < 0.001, ∗∗∗∗*P* < 0.0001 by one-way or two-way ANOVA with Tukey's multiple-comparison test. **Abbreviations**: UMAP, uniform manifold approximation and projection; GO, Gene Ontology; DEGs, differentially expressed genes; SCENIC, single-cell regulatory network inference and clustering; AUCell, area under the curve cell-level scoring; C/EBPβ, CCAAT/enhancer-binding protein beta; mTORC1, mechanistic target of rapamycin complex 1; BMDM, bone marrow–derived macrophage; Ad, adenovirus; RT-qPCR, reverse transcription quantitative polymerase chain reaction; OCR, oxygen consumption rate; FCCP, carbonyl cyanide-p-trifluoromethoxyphenylhydrazone; SEM, standard error of the mean; ANOVA, analysis of variance.

To resolve the differentiation hierarchy of cardiac macrophages, pseudotime analysis (Monocle3) was applied to monocyte–macrophage clusters. A bifurcating trajectory emerged from *Plac8*^+^ monocytes, diverging toward either Cxcl9^+^ inflammatory macrophages or Spp1^+^ reparative macrophages (Fig. 4A and B). Rapamycin treatment remodeled this landscape, markedly restricting monocyte progression into the Cxcl9^+^ lineage (Fig. S3A–C). Along pseudotime, Cxcl9^+^ differentiation was accompanied by progressive induction of interferon-stimulated and pro-inflammatory genes, enriched in pathways of leukocyte activation and cytokine signaling (Fig. 4C and D). In contrast, the Spp1^+^ branch showed gradual upregulation of lipid metabolism, phagocytic, and extracellular matrix–related transcripts, consistent with a reparative phenotype (Fig. S3D–E).

To identify transcriptional regulators governing macrophage lineage differentiation, SCENIC analysis was applied to reconstruct regulon activity across macrophage subsets, revealing 16 regulons with subset-specific activation patterns (Fig. 4E). Comparison among experimental groups demonstrated that inflammatory regulons—including *Cebpb*, *Irf1*, *Hif1a*, and *Nfkb1*—were strongly induced in EAM and significantly downregulated by rapamycin (Fig. S3F), indicating a rapamycin-sensitive regulatory program. Binary regulon activity mapping further showed that *Cebpb* activation was highly restricted to the Cxcl9^+^ macrophage subset while remaining minimal in Lyve1^+^ and Spp1^+^ populations (Fig. 4F), suggesting a lineage-specific role for C/EBPβ. Integration of pseudotime-dependent genes with the Cxcl9^+^ regulon network identified ten shared transcriptional regulators (Fig. 4G), among which Cebpb displayed the highest activity and specificity (Fig. 4H and I). Single-cell expression analysis confirmed upregulation of *Cebpb* in EAM Cxcl9^+^ macrophages, which was significantly reduced following rapamycin treatment (Fig. 4J). Along the Plac8^+^ monocyte–to–Cxcl9^+^ macrophage trajectory, *Cebpb* expression increased progressively, establishing its role as a differentiation-associated regulator (Fig. 4K). Predicted downstream targets of C/EBPβ – including *Traf1*, *Osm*, *Cd44*, *Tlr2* and *Wnt6* – were enriched in immune activation pathways (Fig. 4L and M), supporting a central regulatory function in pathogenic macrophage programming.

To validate the mechanistic link between C/EBPβ activity and mTORC1 signaling, biochemical and functional assays were conducted. Co-immunoprecipitation confirmed the interaction between C/EBPβ and the mTORC1 component Raptor, with C/EBPβ phosphorylation markedly increased in EAM cardiac macrophages and attenuated by rapamycin (Fig. 4N). Functionally, adenoviral-mediated overexpression of *Cebpb* (*Cebpb*-OE) in bone marrow–derived macrophages (BMDMs) induced a Cxcl9^+^-like transcriptional program, characterized by upregulation of *Il6, Tnf,* and *Cxcl9* expression, activation of glycolytic (*Hk2, Pfkm*) and senescence (*Cdkn2a*) genes, and suppression of oxidative phosphorylation—all of which were reversed by rapamycin treatment (Fig. 4O). Seahorse assays further confirmed that *Cebpb*-OE impaired mitochondrial respiration, reducing basal and maximal OCR and ATP production, which were restored by rapamycin (Fig. 4P). Consistently, at the protein level, *Cebpb*-OE markedly increased IL-6, TNF-α, and CXCL9 secretion, and this effect was abolished by rapamycin (Fig. S3G).

Collectively, these findings establish C/EBPβ as a lineage-defining transcriptional regulator that drives the pathogenic differentiation of Plac8^+^ monocytes into Cxcl9^+^ inflammatory macrophages and orchestrates their glycolytic, pro-inflammatory, and senescence-associated reprogramming via mTORC1 signaling. Rapamycin disrupts this mTORC1–C/EBPβ axis, thereby restraining inflammatory macrophage lineage commitment and restoring cardiac immune homeostasis in EAM.

### 3.5 Rapamycin protects cardiomyocytes by disrupting OSM-mediated Macrophage–Cardiomyocyte crosstalk

**Fig. 5:**
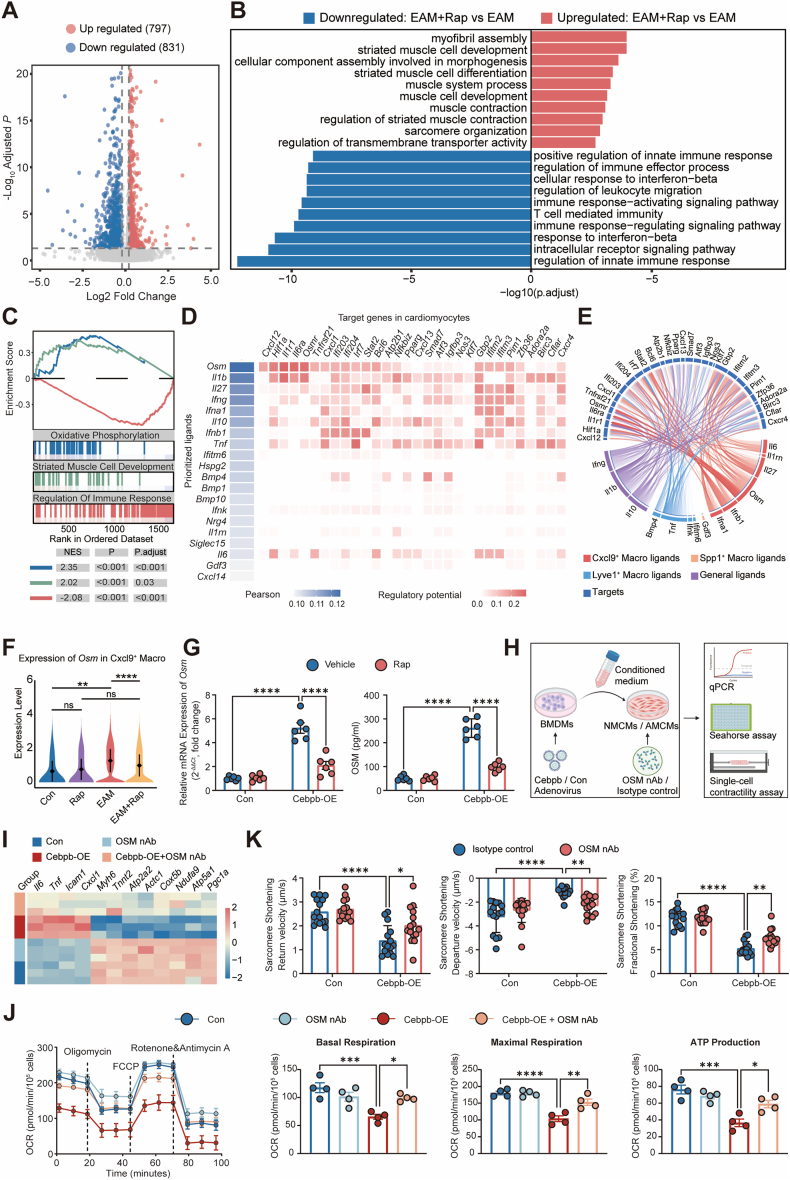
**Macrophage–cardiomyocyte crosstalk via OSM and its modulation by rapamycin.** **A**, Volcano plot of DEGs in Langendorff-isolated cardiomyocytes comparing EAM + rapamycin versus EAM. **B**, GO enrichment of up- and down-regulated DEGs in cardiomyocytes. **C**, GSEA running-score plots for oxidative phosphorylation, striated muscle/cardiac muscle development, and regulation of immune response in cardiomyocytes. **D**, NicheNet-based macrophage to cardiomyocyte communication. Left, ranked ligand activity for macrophage-derived ligands; right, heatmap of predicted regulatory potential of ligand-specific target genes in cardiomyocytes. **E**, Chord diagram visualizing predicted interactions between macrophage subsets and cardiomyocyte targets. **F**, Violin plots showing *Osm* expression in Cxcl9^+^ macrophages across groups. **G**, Validation of OSM in BMDMs. Left, *Osm* mRNA expression measured by RT-qPCR in control and Cebpb-overexpressing (Cebpb-OE) BMDMs treated with vehicle or rapamycin. Right, OSM protein levels in BMDM culture supernatants quantified by ELISA under the same conditions (n = 6 per group). **H**, Schematic illustration of the conditioned medium (CM) co-culture system showing experimental design: BMDMs transduced with control or Cebpb-overexpressing adenovirus were cultured to generate CM, which was subsequently applied to NMCMs or AMCMs in the presence of either isotype control or OSM nAb. Downstream assays included RT-qPCR, Seahorse metabolic analysis, and single-cell contractility measurements. **I**, Heatmap of RT-qPCR results showing relative expression of inflammatory, contractile, and mitochondrial oxidative phosphorylation genes in NMCMs after exposure to CM with or without OSM nAb (n = 4/group). **J**, Seahorse extracellular flux analysis of NMCMs treated as indicated: representative OCR traces following sequential oligomycin, FCCP, and rotenone/antimycin A injections, with bar graphs summarizing basal respiration, maximal respiration, and ATP production (n = 4/group). **K**, Quantification of sarcomere shortening velocity, displacement amplitude, and fractional shortening in single-cell contractility assays of AMCMs exposed to CM, with or without OSM nAb (≥15 cells from 3 hearts/group). Data are presented as mean ± SEM. ns indicates no significant difference, ∗*P* < 0.05, ∗∗*P* < 0.01, ∗∗∗*P* < 0.001, ∗∗∗∗*P* < 0.0001 by one-way or two-way ANOVA with Tukey's multiple-comparison test. **Abbreviations**: DEGs, differentially expressed genes; EAM, experimental autoimmune myocarditis; GO, Gene Ontology; GSEA, gene set enrichment analysis; OSM, oncostatin M; BMDM, bone marrow–derived macrophage; qPCR, quantitative polymerase chain reaction; ELISA, enzyme-linked immunosorbent assay; CM, conditioned medium; NMCM, neonatal mouse cardiomyocyte; AMCM, adult mouse cardiomyocyte; OSM nAb, oncostatin M–neutralizing antibody; RT-qPCR, reverse transcription quantitative polymerase chain reaction; OCR, oxygen consumption rate; FCCP, carbonyl cyanide-p-trifluoromethoxyphenylhydrazone; SEM, standard error of the mean; ANOVA, analysis of variance.

To investigate the impact of Cxcl9^+^ macrophage activity and rapamycin treatment on cardiomyocytes in EAM, bulk RNA sequencing was performed on cardiomyocytes isolated via the Langendorff perfusion system [24]. Comparison between the EAM and EAM + rapamycin groups identified 1628 differentially expressed genes (797 upregulated, 831 downregulated) following rapamycin treatment (Fig. 5A). Functional enrichment revealed two dominant patterns (Fig. 5B): downregulated genes were primarily related to immune and inflammatory responses, including type I interferon signaling and leukocyte activation, whereas upregulated genes were enriched in sarcomere organization, muscle contraction, and myofibril assembly, reflecting improved cardiomyocyte structural and metabolic function. GSEA confirmed that rapamycin enhanced oxidative phosphorylation and muscle development (NES = 2.35, 2.02) while suppressing immune response pathways (NES = −2.08) (Fig. 5C).

To define macrophage–cardiomyocyte communication, NicheNet analysis predicted ligand–target regulatory interactions (Fig. 5D). Several macrophage-derived cytokines exhibited regulatory potential, among which Oncostatin M (OSM) showed the highest ligand activity. OSM-associated cardiomyocyte targets included genes related to inflammatory signaling (*Cxcl12, Il1r1, Il6ra, Osmr*), cellular stress response (*Atf3, Nos3, Bcl6*), and mitochondrial regulation (*Atp2b1, Pparg*), indicating coordinated modulation of inflammatory and metabolic pathways during EAM. To corroborate the predicted OSM–OSMR axis, *Osmr* mRNA levels were quantified by RT-qPCR in isolated cardiomyocytes, revealing increased *Osmr* expression in EAM hearts with partial attenuation following rapamycin treatment (Fig. S4). Chord diagram analysis (Fig. 5E) further revealed that Cxcl9^+^ macrophages were the predominant OSM source, showing extensive predicted interactions along the OSM–OSMR axis with cardiomyocyte inflammatory and metabolic genes. Consistent with these predictions, OSM expression was significantly increased in Cxcl9^+^ macrophages during EAM and suppressed by rapamycin (Fig. 5F). In vitro, *Cebpb*-OE in BMDMs increased *Osm* mRNA and elevated OSM protein in the culture supernatant (Fig. 5G). In parallel, SCENIC regulon analysis identified *Osm* as a predicted C/EBPβ target in Cxcl9^+^ macrophages (Fig. 4L). Together, these data support a model in which C/EBPβ acts upstream of OSM in Cxcl9^+^ macrophages.

To assess the functional impact of macrophage-derived OSM on cardiomyocytes, a conditioned medium co-culture system was established (Fig. 5H). Neonatal mouse cardiomyocytes (NMCMs) treated with conditioned medium from *Cebpb*-OE BMDMs exhibited increased expression of inflammatory genes, reduced contractile gene expression, and suppressed mitochondrial oxidative phosphorylation genes, all of which were ameliorated by OSM-neutralizing antibody (OSM-nAb) (Fig. 5I). Seahorse assays further confirmed that conditioned medium from *Cebpb*-OE BMDMs impaired cardiomyocyte mitochondrial respiration, with reduced ATP production and spare respiratory capacity, effects reversed by OSM blockade (Fig. 5J). Moreover, adult mouse cardiomyocytes (AMCMs) isolated using the Langendorff perfusion system and treated with conditioned medium from *Cebpb*-OE macrophages exhibited significantly impaired contractile performance, as evidenced by reduced sarcomere shortening velocity, decreased peak displacement, and lower fractional shortening in single-cell contraction assays. These contractile defects were effectively reversed by OSM neutralization with OSM-nAb (Fig. 5K).

Collectively, these results identify OSM as a critical effector downstream of C/EBPβ in Cxcl9^+^ macrophages, mediating pathogenic macrophage–cardiomyocyte crosstalk in EAM. Rapamycin protects cardiomyocytes by suppressing the mTOR–C/EBPβ–OSM axis in Cxcl9^+^ macrophages, thereby preserving mitochondrial function, reducing inflammation, and maintaining contractile capacity.

### 3.6 Therapeutic neutralization of OSM attenuates myocardial inflammation and fibrosis and preserves cardiac function in EAM

**Fig. 6:**
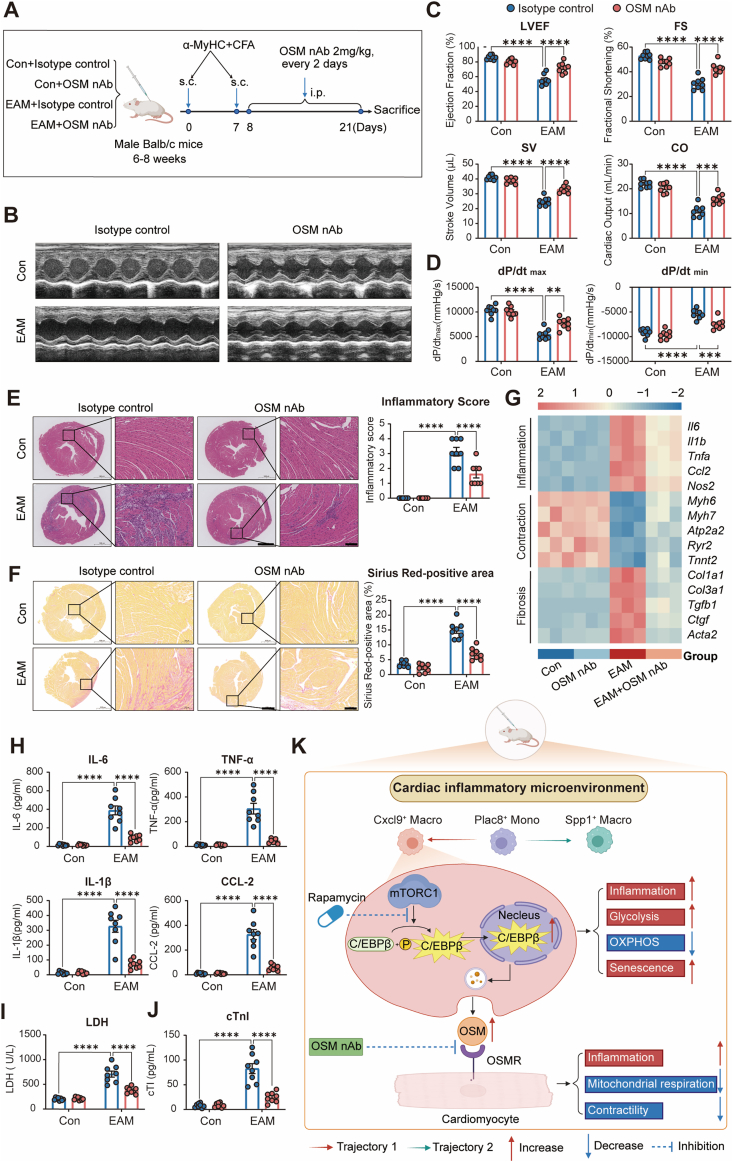
**Therapeutic neutralization of OSM in vivo improves cardiac structure and function in EAM. A**, Schematic of the experimental design. Male BALB/c mice (6–8 weeks) were immunized with α-MyHC/CFA on days 0 and 7 to induce EAM. From day 9 to day 21, mice received intraperitoneal OSM-neutralizing antibody (OSM nAb, 2 mg kg^−1^, every other day) or isotype control; hearts and plasma were collected on day 21. **B**, Representative M-mode echocardiograms in control and EAM mice treated with isotype control or OSM nAb. **C**, Echocardiographic quantification of systolic function: EF, SV, CO, and FS (n = 8/group). **D**, Hemodynamic assessment by Millar catheterization showing peak instantaneous rates of LV pressure rise (dP/dt_max_) and decline (dP/dt_min_) (n = 8/group). **E**, Representative H&E staining of short-axis sections and higher-magnification insets (left) and quantitative inflammatory score (right) (n = 8/group). Scale bars, 1 mm (whole section) and 100 μm (insets). **F**, Representative Sirius Red staining (left) and quantification of collagen deposition (right) in the indicated groups (n = 8/group). Scale bars, 1 mm (whole section) and 100 μm (insets). **G**, Heatmap of cardiac mRNA expression (detected by RT-qPCR) for representative inflammatory, fibrotic, contractile, and mitochondrial genes from control and EAM hearts treated with isotype control or OSM nAb (n = 8/group). **H**, Plasma pro-inflammatory cytokines (IL-6, TNF-α, IL-1β, CCL2) measured by ELISA (n = 8/group). **I** and **J**, Circulating cardiac injury markers [LDH (**I**) and cardiac troponin I (**J**)] levels detected by the reagent kit (n = 8/group). **K**, Schematic model of this study. Rapamycin restrains mTORC1–C/EBPβ signaling to limit Cxcl9^+^ inflammatory macrophage activation and metabolic reprogramming, while promoting reparative Spp1^+^ macrophage states. Macrophage-derived OSM drives cardiomyocyte dysfunction via OSM–OSMR signaling, which is attenuated by OSM neutralization. Data are presented as mean ± SEM; ∗*P* < 0.05, ∗∗*P* < 0.01, ∗∗∗*P* < 0.001, ∗∗∗∗*P* < 0.0001 by two-way ANOVA with Tukey's multiple-comparison test. **Abbreviations**: α-MyHC, α-myosin heavy chain; CFA, complete Freund's adjuvant; EAM, experimental autoimmune myocarditis; OSM, oncostatin M; nAb, neutralizing antibody; EF, ejection fraction; SV, stroke volume; CO, cardiac output; FS, fractional shortening; dP/dt_max_, maximum rate of left ventricular pressure rise; dP/dt_min_, maximum rate of left ventricular pressure decline; H&E, hematoxylin and eosin; RT-qPCR, reverse transcription quantitative PCR; IL, interleukin; TNF-α, tumor necrosis factor-α; CCL2, chemokine (C–C motif) ligand 2; ELISA, enzyme-linked immunosorbent assay; LDH, lactate dehydrogenase; ANOVA, analysis of variance; SEM, standard error of the mean.

To further validate the pathogenic role of Cxcl9^+^ macrophage-derived OSM in vivo, we administered an OSM-nAb in EAM mice following the regimen illustrated in Fig. 6A. Echocardiographic evaluation revealed that OSM blockade significantly preserved cardiac systolic function, as evidenced by improved LVEF and FS, compared to isotype controls (Fig. 6B–C). Hemodynamic assessment using Millar catheterization further demonstrated that OSM neutralization restored dp/dt_max_ and dp/dt_min_ values, indicating improved ventricular contractility and relaxation (Fig. 6D).

Histological analyses corroborated these functional improvements. H&E staining showed a marked reduction in myocardial inflammatory infiltration following OSM blockade (Fig. 6E), while Sirius Red staining confirmed attenuation of myocardial fibrosis (Fig. 6F). Consistently, qPCR analysis of cardiac tissue revealed that OSM nAb treatment suppressed the expression of inflammatory cytokines, fibrosis markers, and restored contractile and mitochondrial genes (Fig. 6G). Systemically, plasma ELISA assays demonstrated that OSM blockade significantly reduced circulating levels of pro-inflammatory cytokines IL-6, TNF-α, IL-1β, and CCL2 in EAM mice (Fig. 6H). Furthermore, cardiac injury biomarkers including plasma LDH and cTnI were markedly elevated in EAM mice and significantly decreased upon OSM neutralization, reflecting alleviated myocardial damage (Fig. 6I and J).

Collectively, these data validate OSM as a central downstream effector of pathogenic macrophage–cardiomyocyte signaling in EAM. In vivo OSM neutralization alleviates myocardial inflammation, limits fibrotic remodeling, and preserves systolic performance.

## 4 Discussion

In this study, we delineate a mechanistic framework in which rapamycin preserves cardiac function and mitigates inflammatory and fibrotic remodeling in EAM by reprogramming cardiac macrophage states. Single-cell transcriptomics identified monocyte–macrophages as the principal rapamycin-responsive compartment, wherein mTORC1 inhibition suppressed glycolytic, inflammatory, and senescence programs while restoring oxidative metabolism. Within this population, a Cxcl9^+^ macrophage subset emerged as the dominant pathogenic driver, orchestrated by an mTORC1–C/EBPβ axis and communicating with cardiomyocytes through OSM. Neutralization of OSM in vivo reproduced much of rapamycin's protective phenotype, nominating the mTORC1–C/EBPβ–OSM pathway as a central therapeutic axis in myocarditis and chronic inflammatory cardiomyopathy.

While advances in intensive care and immunomodulatory therapies have reduced acute mortality from myocarditis, a substantial proportion of survivors progress to chronic inflammatory cardiomyopathy characterized by persistent low-grade inflammation, ventricular dilation, and fibrotic remodeling [11,25,26]. This clinical trajectory underscores the need for strategies targeting the immune–metabolic checkpoints sustaining post-inflammatory injury. Notably, the recent HYPIC phase II trial demonstrated that hydroxychloroquine, an immunomodulatory quinoline derivative, significantly reduced major adverse cardiovascular events in patients with chronic inflammatory cardiomyopathy following fulminant myocarditis, reinforcing the therapeutic potential of immune-metabolic interventions [8]. Consistent with this paradigm, our data reveal that rapamycin, a selective mTORC1 inhibitor, preserves systolic function and ameliorates structural injury when administered during the inflammatory phase of EAM. Echocardiographic and hemodynamic improvements were accompanied by marked reductions in histologic inflammation, collagen deposition, and systemic cytokine release. At single-cell resolution, rapamycin selectively suppressed the expansion and activation of cardiac monocyte–macrophages, which represented the most abundant immune subset in EAM [27,28]. Gene set enrichment and metabolic flux analyses confirmed a shift from glycolysis-coupled inflammation toward restored oxidative phosphorylation, suggesting that mTORC1 inhibition realigns macrophage bioenergetics toward a reparative phenotype. These findings align with prior reports demonstrating that mTORC1 activity drives pro-inflammatory polarization and metabolic dysfunction in macrophages [[29], [30], [31]]. It should be acknowledged that prolonged rapamycin exposure can, in some settings, secondarily modulate mTORC2-dependent signaling. In our study, pathway scoring analysis did not reveal a consistent activation of an mTORC2-related gene signature in cardiac macrophages across conditions, nor a major rapamycin-induced shift; however, we did not systematically quantify canonical mTORC2 markers such as Akt Ser473 phosphorylation. Thus, although our functional and transcriptional data converge on mTORC1 as the dominant metabolic checkpoint in pathogenic cardiac macrophages, a contributory role of mTORC2 cannot be formally excluded and warrants dedicated investigation in future work.

Interferon-responsive CXCL9^+^/CXCL10^+^ macrophages have emerged as a conserved pro-inflammatory myeloid subset in diverse cardiac injuries, including immune checkpoint inhibitor myocarditis and viral myocarditis [[32], [33], [34]]. Single-cell atlases have shown that these CCR2^+^, IFN-γ–driven macrophages amplify T-cell recruitment and cytokine signaling, and their depletion mitigates cardiac injury [33,35]. Our data identify a similar interferon-high Cxcl9^+^ population as the principal inflammatory macrophage subset in EAM, exhibiting dominant enrichment in inflammatory and senescence gene modules. Pseudotime reconstruction revealed a trajectory from Plac8^+^ monocytes bifurcating into either Cxcl9^+^ inflammatory or Spp1^+^ reparative fates. Notably, rapamycin restricted monocyte differentiation toward the Cxcl9^+^ lineage. Regulon analysis positioned C/EBPβ as the highest-ranking transcriptional driver within the Cxcl9^+^ subset, with activity increasing along the monocyte-to-Cxcl9^+^ continuum and suppressed under mTORC1 inhibition. C/EBPβ integrates metabolic and inflammatory inputs and operates downstream of mTORC1 to coordinate macrophage activation, linking nutrient sensing to cytokine output [36,37]. The identification of a C/EBPβ-centered axis provides a mechanistic explanation for how mTORC1 inhibition attenuates interferon-driven macrophage states [38]. These insights expand prior observations that cataloged interferon-high macrophages in myocarditis but did not define their upstream regulatory control. By revealing an mTORC1–C/EBPβ signaling module governing this fate, our work conceptually bridges immune metabolism with chronic inflammation in the myocardium.

OSM, a member of the gp130 cytokine family, exerts context-dependent effects in the heart [39,40]. Transient OSM–OSMR activation can be cardioprotective in acute settings: in myocardial infarction and ischaemia/reperfusion models, short-term OSM signaling promotes cardiomyocyte dedifferentiation with functional recovery, improves post-infarct remodeling, and limits I/R injury by enhancing mitochondrial biogenesis and insulin sensitivity [21,41,42]. By contrast, persistent or excessive OSM/OSMR activation drives maladaptive remodeling and heart failure; sustained OSM signaling is increased in dilated cardiomyopathy and diabetic cardiomyopathy, where it promotes cardiomyocyte dedifferentiation and contractile failure, while genetic or pharmacologic OSMRβ inhibition ameliorates inflammatory heart failure [43]. In addition, macrophage hypoxia–induced OSM can restrain fibroblast activation and fibrosis, further underscoring its biphasic and microenvironment-dependent actions [44]. In our model, OSM was identified as the most active macrophage-derived ligand targeting cardiomyocyte genes involved in inflammation, stress, and mitochondrial function. Mechanistically, co-immunoprecipitation confirmed an interaction between C/EBPβ and the mTORC1 component Raptor, with enhanced C/EBPβ phosphorylation in EAM macrophages that was abrogated by rapamycin. Overexpression of *Cebpb* in bone marrow–derived macrophages reproduced the Cxcl9^+^ signature—upregulating *Il6, Tnf*, and glycolytic genes while impairing oxidative phosphorylation and ATP generation—effects that were partially reversed by mTORC1 inhibition. Conditioned-medium experiments demonstrated that macrophage-derived OSM impaired cardiomyocyte bioenergetics and contractility, while OSM neutralization rescued both mitochondrial respiration and contractile performance. Importantly, systemic blockade of OSM in vivo reproduced rapamycin's cardioprotective effects, improving LVEF, hemodynamic indices, and reducing myocardial inflammation, fibrosis, and circulating cytokines. This functional validation establishes OSM as a pathogenic mediator of macrophage–cardiomyocyte communication. Previous studies have described gp130/OSMR signaling as a double-edged sword—beneficial in acute injury yet detrimental when chronically sustained [[45], [46], [47]]. Our findings identify the immune–metabolic upstream trigger of this maladaptive activation: a C/EBPβ-dependent macrophage circuit regulated by mTORC1. Thus, OSM serves as both a mechanistic effector and a potential downstream therapeutic readout of mTORC1–C/EBPβ activity. Targeting this pathway could provide disease specificity with less systemic immunosuppression compared with direct mTOR inhibition, although careful definition of dose, timing, and disease stage—including future titrated OSM overexpression studies in control and EAM hearts—will be required to fully delineate its therapeutic window.

Several limitations merit consideration. First, the EAM model, while recapitulating autoimmune myocarditis, does not fully reflect the etiologic heterogeneity of human disease, including viral and immune checkpoint inhibitor–associated myocarditis. Second, although our data highlight a dominant myeloid mechanism, rapamycin has pleiotropic effects, and contributions from lymphoid or other non-myeloid compartments cannot be excluded. Third, the mTORC1–C/EBPβ–OSM axis is supported by integrative analyses, *Cebpb* gain-of-function experiments, and OSM neutralization, but we did not perform myeloid-specific *Cebpb* loss-of-function studies; future work with conditional *Cebpb* deletion or selective C/EBPβ inhibitors will be required to establish necessity. Fourth, we focused on mTORC1; although mTORC2 pathway scoring in macrophages did not show consistent activation, canonical mTORC2 readouts (e.g. Akt Ser473) were not systematically quantified. Finally, our in vivo OSM interventions were restricted to neutralization in EAM; dedicated, titrated OSM overexpression and timing studies in control and myocarditis models will be needed to define whether OSM alone can drive cardiac injury and to delineate a safe therapeutic window.

In conclusion, our findings establish a coherent mechanistic model in which mTORC1 inhibition reprograms monocyte–macrophages away from a C/EBPβ-driven Cxcl9^+^ inflammatory fate, thereby disrupting OSM-mediated macrophage–cardiomyocyte signaling that compromises mitochondrial and contractile function. The mTORC1–C/EBPβ–OSM axis thus defines a critical immune–metabolic pathway sustaining myocardial inflammation and represents a tractable therapeutic target for preventing the progression of autoimmune myocarditis to chronic inflammatory cardiomyopathy.

## Funding

The present study was supported, in part, by projects from the National Natural Science Foundation of China (Nos. 82241034 and 82330010 for DWW), the Top-Notch Talent Program of Hubei Province and Tongji Hospital (No. 2021YBJRC005 for DWW), the Hubei Provincial Key Laboratory Program (No. 2025CSA042 for DWW), and the Scientific Research Project of Hubei Province (Nos. 2024CSA067 and 2025CSA042 for DWW), and by the Key R&D Program of the Department of Science and Technology of Hubei Province, China (No. 2023BCB013 for XQR).

## CRediT authorship contribution statement

**Yan Zhuang:** Data curation, Investigation, Writing – original draft, Writing – review & editing. **Yongcui Yan:** Investigation, Methodology, Software, Writing – original draft, Writing – review & editing. **Zheng Wen:** Data curation, Methodology. **Xiaoquan Rao:** Data curation, Formal analysis, Methodology. **Jiangang Jiang:** Investigation, Methodology. **Huihui Li:** Supervision, Writing – review & editing. **Dao Wen Wang:** Funding acquisition, Supervision, Writing – original draft, Writing – review & editing.

## Declaration of competing interest

The authors declare that they have no known competing financial interests or personal relationships that could have appeared to influence the work reported in this paper.

## Data Availability

Data will be made available on request.
