# Supplementary material for: Rapamycin preserves cardiac function in autoimmune myocarditis by reprogramming Cxcl9+ macrophages via the mTORC1–C/EBPβ–OSM axis
Source: Redox Biol. 2025 Dec 13;89:103970. doi: 10.1016/j.redox.2025.103970 (PMC12771342; doi:10.1016/j.redox.2025.103970)
Supplement: Multimedia component 1 [file mmc1.docx]

# **Supplemental Materials**

**Supplementary methods**

**Animal Models and Treatments**

**Establishment of Experimental Autoimmune Myocarditis (EAM) Model**

Male BALB/c mice (6–8 weeks old; ~18–22 g) were purchased from GemPharmatech (Nanjing, China) and housed under specific-pathogen-free conditions with a 12-h light/dark cycle and free access to food and water. After acclimation for one week, mice were randomly assigned to experimental groups. To induce EAM, mice received subcutaneous immunizations on days 0 and 7 at bilateral flank sites with 100 µg of α-myosin heavy chain peptide (α-MyHC) emulsified in an equal volume of Complete Freund’s Adjuvant (CFA; Sigma-Aldrich, Cat. No. F5881), in a total volume of 100 µL per mouse (50 µL per flank). Control mice were injected with CFA alone (vehicle) in the same schedule. This immunization protocol was adapted from established EAM models to elicit cardiac-specific autoimmune inflammation. [1]

**Rapamycin and OSM-neutralizing Antibody Interventions**

Beginning from day 8 post-immunization (onset of inflammatory phase), mice in the Rap and EAM+Rap groups received rapamycin (Sigma-Aldrich, Cat. No. R0395) dissolved in vehicle (10% PEG-400, 5% Tween-80, 85% saline) delivered intraperitoneally at 1 mg/kg body weight every other day until day 20. This dosing regimen (1 mg/kg i.p.) was selected based on prior cardiovascular and immunologic studies demonstrating reliable mTORC1 inhibition with favorable tolerability in murine chronic inflammatory models.[2,3]

For OSM neutralization, mice in the EAM+OSM-nAb group received anti-mouse OSM monoclonal antibody (R&D Systems, clone MAB4951, Cat. No. MAB4951) intraperitoneally at 2 mg/kg every other day from day 9 to day 21. An isotype-matched rat IgG2B control antibody (same schedule and dose) was administered in parallel in the EAM and EAM+Rap groups. All antibody reagents were reconstituted and sterile-filtered per manufacturer instructions before injection.

At day 21, mice were humanely euthanized for tissue harvest (hearts, blood) and downstream analyses (functional, histological, molecular, cellular). All animal experiments were performed in a randomized and investigator-blinded manner. Sample size was determined based on prior studies and pilot experiments. All procedures were conducted in accordance with institutional guidelines for animal care and approved by by the Institutional Animal Care and Use Committee of Tongji Medical College, Huazhong University of Science and Technology (approval No. 4364).

**Cardiac Functional Assessment**

**Transthoracic Echocardiography**

Left ventricular systolic function was evaluated by transthoracic echocardiography using a high-resolution ultrasound system (Vevo 1100, VisualSonics, Toronto, Canada) equipped with a 30-MHz linear array transducer. Mice were lightly anesthetized with 1.0–1.5% isoflurane in 100% oxygen to maintain heart rates between 450–550 beats/min while avoiding cardiodepression. Body temperature was maintained at 37 °C using a heated platform. Parasternal long-axis and short-axis images were acquired in B-mode, and M-mode tracings were recorded at the papillary muscle level. Left ventricular ejection fraction (LVEF), fractional shortening (FS), stroke volume (SV), and cardiac output (CO) were calculated from M-mode measurements using standard equations based on the American Society of Echocardiography guidelines. All measurements were averaged from three consecutive cardiac cycles and analyzed offline using Vevo LAB software (v3.2.6, VisualSonics) by an investigator blinded to group allocation.

**Hemodynamic Analysis**

Invasive hemodynamic measurements were performed using a conductance-based pressure–volume (PV) catheter system (Millar SPR-835, 1.4F; Millar Instruments, Houston, TX, USA). Mice were anesthetized with 2% isoflurane, intubated, and mechanically ventilated. Following median sternotomy, the PV catheter was advanced retrogradely into the left ventricle via the right carotid artery. Real-time ventricular pressure–volume loops were acquired at steady state using PowerLab/Chart software (ADInstruments) and calibrated using parallel conductance and cuvette calibration methods. Hemodynamic indices including maximum rate of pressure rise (dP/dt_max_) and maximum rate of pressure decay (dP/dt_min_) were derived using LabChart Pro software (v8.1, ADInstruments). Data were averaged from at least 10 consecutive cardiac cycles under stable hemodynamic conditions, and analysis was performed in a blinded manner.[4]

**Histological and Immunohistochemical Analysis**

**Tissue Preparation and Histochemical Staining**

After euthanasia, hearts were excised, rinsed in cold phosphate-buffered saline (PBS), and fixed overnight in 4% neutral-buffered formalin at 4 °C. Tissues were then dehydrated, cleared, and embedded in paraffin. Serial transverse sections (4 μm thickness) were cut through the mid-ventricular level. For assessment of inflammation, sections were stained with hematoxylin and eosin (H&E) following standard protocols. To visualize collagen deposition and fibrotic remodeling, sections were stained with Picrosirius Red / Sirius Red (Direct Red 80; Sigma-Aldrich Cat. No. 365548) in saturated picric acid solution (0.1% w/v) for 60 minutes, followed by two washes in acidified water (0.5% acetic acid), dehydration, clearing, and mounting. Under bright-field microscopy, collagen fibers appear red against a pale background; polarization microscopy may be used to enhance collagen birefringence. Quantitative morphometric analysis of inflammatory area and fibrotic collagen percentage was performed using ImageJ on at least 5 randomly selected high-power fields per section per heart, by investigators blinded to group assignment.

**Immunofluorescence Staining and Imaging**

Paraffin sections were deparaffinized, rehydrated, and subjected to antigen retrieval (citrate buffer, pH 6.0, 95 °C for 10 minutes). Endogenous autofluorescence and background were blocked with 5% bovine serum albumin plus 0.3% Triton X-100 for 30 minutes at room temperature. Sections were incubated overnight at 4 °C with primary antibodies including anti-mouse F4/80 (ABclonal #A23788), anti-CXCL9 (Abcam #ab60030), at empirically determined dilutions in blocking buffer. After PBS washes, sections were incubated with species-appropriate fluorescent secondary antibodies (Alexa Fluor 488/594; Thermo Fisher) for 1 hour at room temperature, and nuclear counterstaining was performed with DAPI (4′,6-diamidino-2-phenylindole). Slides were mounted with anti-fade medium. Imaging was conducted on a laser-scanning confocal microscope (Zeiss LSM 880) using identical acquisition settings across groups. Colocalization and fluorescence quantification were performed using ImageJ on three non-overlapping fields per section, averaged for each mouse, under acquisition-blinded conditions.

**Molecular and Biochemical Assays**

**Quantitative Real-Time PCR (qPCR)**

Heart tissues or sorted cell pellets were homogenized in TRIzol reagent (Invitrogen, Cat. No. 15596018), and total RNA was extracted following the manufacturer’s protocol. RNA purity and concentration were assessed by NanoDrop 2000 (Thermo Fisher). cDNA was synthesized using PrimeScript RT Master Mix (Takara, Cat. No. RR036A) with 1 µg of RNA in a 20 µL reaction. Quantitative PCR was performed on a Bio-Rad CFX96 real-time PCR system using SYBR Green PCR Master Mix (ABclonal, Cat. No. RK21203) under cycling conditions: 95 °C for 3 min; 40 cycles of 95 °C for 10 s, 60 °C for 30 s; followed by melt-curve analysis to confirm single specificity. Primer sequences (forward and reverse) for target genes were designed using PrimerBank or literature-validated sources; β-actin served as the internal reference gene. Relative expression levels were calculated using the comparative ΔΔCt method. The primers for qRT-PCR were as follows:

| **Primer Name** | **Forward Sequence** | **Reverse Sequence** |
| --- | --- | --- |
| Il-6 | 5’-CTGCAAGAGACTTCCATCCAG-3’ | 5’-AGTGGTATAGACAGGTCTGTTGG-3’ |
| Tnf-α | 5’-CAGGCGGTGCCTATGTCTC-3’ | 5’-CGATCACCCCGAAGTTCAGTAG-3’ |
| Il-1β | 5’-GAAATGCCACCTTTTGACAGTG-3’ | 5’-TGGATGCTCTCATCAGGACAG-3’ |
| Ccl2 | 5’-TTCTTCGATTTGGGTCTCCTTG-3’ | 5’-GTGCAGCTCTTGTCGGTGAA-3’ |
| Nos2 | 5’-CTGCAAGCACAATGGGGAGT-3’ | 5’-CGTCGGTAGAGAGACTGCTG-3’ |
| Myh6 | 5’-GCCCAGTACCTCCGAAAGTC-3’ | 5’-ATCAGGCACGAAGCACTCC-3’ |
| Myh7 | 5’-CATGGGATGGTAAGAAACGGG-3’ | 5’-TCCTCCAGTAAGTCGAAACGG-3’ |
| Atp2a2 | 5’-GAGAACGCTCACACAAAGACC-3’ | 5’-ACTGCTCAATCACAAGTTCCAG-3’ |
| Ryr2 | 5’-GCCACCGGACACTCCTCTAT-3’ | 5’-CCAACACGCACTTTTTCTCCT-3’ |
| Tnnt2 | 5’-CAGAGGAGGCCAACGTAGAAG-3’ | 5’-CTCCATCGGGGATCTTGGGT-3’ |
| Col1a1 | 5’-GCTCCTCTTAGGGGCCACT-3’ | 5’-ATTGGGGACCCTTAGGCCAT-3’ |
| Col3a1 | 5’-CTGTAACATGGAAACTGGGGAAA-3’ | 5’-CCATAGCTGAACTGAAAACCACC-3’ |
| Tgfb1 | 5’-ATGTCACGGTTAGGGGCTC-3’ | 5’-GGCTTGCATACTGTGCTGTATAG-3’ |
| Ctgf | 5’-GGCCTCTTCTGCGATTTCG-3’ | 5’-GCAGCTTGACCCTTCTCGG-3’ |
| Acta2 | 5’-CCCAGACATCAGGGAGTAATGG-3’ | 5’-TCTATCGGATACTTCAGCGTCA-3’ |
| Cxcl9 | 5’-GGAGTTCGAGGAACCCTAGTG-3’ | 5’-GGGATTTGTAGTGGATCGTGC-3’ |
| Cdkn2a | 5’-CTTCGCCGAGCAGTTTCGT-3’ | 5’-TCAATCCCATCAGCCATTTCC-3’ |
| Hk2 | 5’-ATGATCGCCTGCTTATTCACG-3’ | 5’-CGCCTAGAAATCTCCAGAAGGG-3’ |
| Pfkm | 5’-CATCGCCGTGTTGACCTCT-3’ | 5’-CCCGTGAAGATACCAACTCGG-3’ |
| Cox5b | 5’-GGAAGACCCTAATCTAGTCCCG-3’ | 5’-GTTGGGGCATCGCTGACTC-3’ |
| Ndufa9 | 5’-GTCCGCTTTCGGGTTGTTAGA-3’ | 5’-CCTCCTTTCCCGTGAGGTA-3’ |
| Icam1 | 5’-GTGATGCTCAGGTATCCATCCA-3’ | 5’-CACAGTTCTCAAAGCACAGCG-3’ |
| Cxcl1 | 5’-TCGAGACCATTTACTGCAACAG-3’ | 5’-CATTGCCGGTGGAAATTCCTT-3’ |
| Actc1 | 5’-CTGGATTCTGGCGATGGTGTA-3’ | 5’-CGGACAATTTCACGTTCAGCA-3’ |
| Atp5a1 | 5’-TCTCCATGCCTCTAACACTCG-3’ | 5’-CCAGGTCAACAGACGTGTCAG-3’ |
| Pgc1a | 5’-TATGGAGTGACATAGAGTGTGCT-3’ | 5’-GTCGCTACACCACTTCAATCC-3’ |
| β-actin | 5’-CAACGGCTCCGGCATGTG-3’ | 5’-GTCCTTCTGACCCATTCCCA-3’ |

**Enzyme-Linked Immunosorbent Assay (ELISA)**

Plasma was collected by retro-orbital or cardiac puncture into EDTA-coated tubes, centrifuged (1,500 × g, 15 min, 4 °C), and supernatants stored at –80 °C. Concentrations of cytokines IL-6 and TNF-α were quantified using Quantikine Mouse IL-6 (Cat. No. M6000B) and Mouse TNF-α (Cat. No. MTA00B) ELISA kits (R&D Systems) according to manufacturer instructions. Matrix-matched standards and samples were measured in duplicate; absorbance was read at 450 nm with wavelength correction at 570 nm. For cardiac injury markers, plasma LDH activity was measured using a colorimetric LDH assay kit (Abcam, Cat. No. ab102526) and cardiac troponin I (cTnI) was assessed by a high-sensitivity mouse cTnI ELISA (ABclonal, Cat. No. RK04850). Sample values were interpolated from calibration curves and normalized per manufacturer protocols.

**Western Blotting**

Cardiac macrophages isolated via F4/80 magnetic sorting were lysed in RIPA buffer (50 mM Tris-HCl pH 7.4, 150 mM NaCl, 1% NP-40, 0.1% SDS, 1 mM EDTA) supplemented with protease and phosphatase inhibitors (Abclonal, Cat. No. RM02997). Protein concentrations were measured by BCA assay (Abclonal, Cat. No. BRK0011). Equal amounts (20–30 µg) of protein were separated by SDS-PAGE (10%–12% gels) and transferred to PVDF membranes (Millipore). Membranes were blocked with 5% non-fat milk in TBS-T (0.1% Tween-20) for 1 h at room temperature and incubated overnight at 4 °C with primary antibodies: anti–p-mTOR (Ser2448) (Cell Signaling Technology, Cat. No. 5536), total mTOR (CST #2983), p-S6 (CST #2215), HK2 (CST #2867), mt-ND4 (Abcam #ab74214), NF-κB1 (CST #3033), CDKN1A/p21 (Abclonal #A21897), and β-actin (Abclonal #AC026). Following washes, membranes were incubated with HRP-conjugated secondary antibodies (anti-rabbit or anti-mouse IgG-HRP, CST) for 1 h. Protein bands were visualized using enhanced chemiluminescence (ECL; Thermo Fisher) and imaged by ChemiDoc MP (Bio-Rad). Densitometric quantification was performed using ImageJ software, normalized to β-actin or corresponding total protein bands, and expressed relative to control samples.

**Isolation of Cardiac Immune Cells and Macrophages**

**Isolation of Cardiac CD45⁺ Immune Cells**

After euthanasia, freshly excised hearts were rapidly perfused with cold PBS to remove residual blood, minced into ~1 mm³ pieces, and enzymatically digested in a buffer containing collagenase II (200 U/mL), collagenase IV (100 U/mL), DNase I (50 U/mL) in HBSS at 37 °C for 30–40 minutes under gentle agitation. The digested suspension was filtered through a 70-µm cell strainer and centrifuged (400 × g, 5 min, 4 °C). Red blood cells were removed by ACK lysis buffer (0.15 M NH_4_Cl, 10 mM KHCO_3_, 0.1 mM EDTA, pH 7.2) for 2 minutes on ice. The remaining cells were washed and resuspended in MACS buffer (PBS + 0.5% BSA + 2 mM EDTA). CD45⁺ leukocytes were enriched by immunomagnetic positive selection using mouse CD45 MicroBeads (Miltenyi Biotec, Cat. No. 130-052-301) on an MS/LS column according to the manufacturer’s protocol. After enrichment, cell viability was assessed using trypan blue exclusion or propidium iodide staining, and only samples with viability > 85% proceeded to downstream single-cell RNA sequencing.[5]

**Isolation of F4/80⁺ Macrophages**

From the CD45-enriched fraction or directly from cardiac single-cell suspensions, F4/80⁺ macrophages were further purified using Anti-F4/80 MicroBeads UltraPure (Miltenyi Biotec, Cat. No. 130-110-443) via positive selection on MACS columns, as described in manufacturer’s protocols. Briefly, cells were incubated with microbeads in MACS buffer for 15 minutes at 4 °C, washed, and passed through MS or LS columns placed in the magnetic field; magnetically labeled F4/80⁺ cells were retained and then eluted after removal from the magnet. Purity of the isolated macrophages was confirmed via flow cytometry using anti-F4/80 and anti-CD11b antibodies; only fractions with >90% F4/80⁺CD11b⁺ cells were used for subsequent immunoblotting and Seahorse metabolic assays.

**Single-Cell RNA Sequencing and Computational Analysis**

**Library Preparation and Sequencing**

Cardiac CD45⁺ immune cells freshly isolated from mouse hearts were processed immediately for single-cell transcriptomic profiling. Cell suspensions were prepared using the SeekMate Tissue Dissociation Reagent Kit A Pro (SeekGene, K01801301) according to the manufacturer’s protocol, followed by red blood cell lysis (Solarbio, R1010) and filtration through 70 µm strainers. Viable cells were counted using a Countstar Rigel S2 fluorescence cell analyzer with AO/PI staining. Single-cell RNA-seq libraries were constructed using the SeekOne® Digital Droplet Single Cell 3′ Library Preparation Kit (SeekGene, K00202). Briefly, barcoded hydrogel beads, partitioning oil, and cell suspensions were loaded onto a SeekOne® Chip S3 to generate droplets. Reverse transcription was carried out at 42 °C for 90 min, followed by emulsion breaking, cDNA amplification, fragmentation, end-repair, A-tailing, adapter ligation, and indexed PCR. The final cDNA libraries were purified with VAHTS DNA Clean Beads (Vazyme, N411-01) and quality-checked using a Qubit Fluorometer (Thermo Fisher, Q33226) and Bio-Fragment Analyzer (Bioptic, Qsep400). Sequencing was performed on an Illumina NovaSeq 6000 or DNBSEQ-T7 platform with a 150 bp paired-end (PE150) read length, achieving an average depth of > 50,000 reads per cell.[6]

**Data Processing**

Raw sequencing data were demultiplexed and aligned to the mouse mm10 genome using Cell Ranger. The resulting feature-barcode matrices were imported into Seurat (v4.3.0) for downstream analyses. Quality control filtering removed cells with < 200 or > 6,000 genes, > 20,000 UMIs, or > 10% mitochondrial gene content. After log-normalization and scaling, the top 2,000 highly variable genes were identified for principal-component analysis (PCA). Batch effects across biological replicates were corrected using the Harmony algorithm. Clustering was performed via shared nearest-neighbor (SNN) modularity optimization at resolutions 0.3–0.8, and cell distributions were visualized using uniform manifold approximation and projection (UMAP). Doublets and ambient RNA contamination were removed through iterative filtering and comparison with established immune reference datasets.

**Cell Annotation and Subclustering**

Major immune lineages were annotated based on canonical marker expression, including Cd68 (macrophages), Ly6g (neutrophils), Cd3e (T cells), and Cd79a (B cells). Monocyte–macrophage clusters were identified by Cd68 and Adgre1 expression and subsequently reclustered to delineate transcriptionally distinct subpopulations, characterized by Lyve1⁺, Plac8⁺, Cxcl9⁺, Spp1⁺, Top2a⁺, and Cd209a⁺ phenotypes. Marker gene identification was performed using the Wilcoxon rank-sum test within Seurat (log₂FC > 0.25, adjusted *P* < 0.05). Cluster-specific expression patterns were visualized by heatmaps, dot plots, and violin plots. Differentially expressed genes (DEGs) between EAM and EAM + rapamycin groups were calculated using the MAST model, controlling for UMI counts and cell cycle phase.

**Downstream Analyses**

Functional enrichment of DEGs was performed using clusterProfiler (v4.6.0) for Gene Ontology (GO) analysis, and Gene Set Enrichment Analysis (GSEA) was applied using the MSigDB v7.5 hallmark gene sets. Metabolic and inflammatory pathway activities—including glycolysis, oxidative phosphorylation, inflammatory response, and senescence—were quantified through gene-set scoring with Seurat’s AddModuleScore function. Odds ratio (OR) analysis for cell-type enrichment across groups was conducted using fisher.test in R. Pseudotime trajectory inference was implemented using Monocle3, transcription-factor regulon analysis with pySCENIC, and ligand–receptor interactions were predicted via NicheNet. All computational analyses were performed in R (v4.3.2) or Python (v3.9) environments on a Linux workstation.

**Trajectory and Regulatory Network Analyses**

**Pseudotime Analysis**

To reconstruct differentiation trajectories of monocyte–macrophage subsets, we utilized Monocle 3 (v1.2.9). The scaled expression matrix and cluster assignments from Seurat (Section 7) were converted into a CellDataSet object. We preprocessed the data via preprocess_cds (num_dim = 50) and aligned for batch effects using align_cds with batch as the alignment group. Cells were clustered with cluster_cells, and reduced dimensional embedding was performed via UMAP. The learn_graph function was then used to infer principal graph topology, and order_cells was applied to define pseudotime ordering, selecting Plac8⁺ monocytes as the root node based on expression of early-monocyte markers. To resolve branching into Cxcl9⁺ versus Spp1⁺ lineages, branch-specific cells were partitioned, and graph_test was used to identify genes whose expression varies along pseudotime or between branches. Differential branch-dependent gene sets were extracted based on Moran’s I q-values < 0.05 and visualized as heatmaps ordered by pseudotime progression.

**Transcription Factor Regulatory Network (SCENIC)**

To infer transcriptional regulons and their activity, we applied the SCENIC workflow via pySCENIC (v0.12.1) on filtered scRNA-seq expression data. First, co-expression modules were detected using GRNBoost2, then cis-regulatory motif enrichment and regulon pruning were conducted with RcisTarget databases (motif rankings for mm10). The activity of each regulon in individual cells was quantified by AUCell, yielding a cell-by-regulon activity matrix. Regulon activity scores were transferred into Seurat objects, aligned with macrophage subclusters, and visualized as UMAP overlays or heatmaps. We specifically examined the activity and specificity of transcription factors including Cebpb, Irf1, Hif1a, and Nfkb1, ranking them by specificity scores (difference in AUC across clusters). Regulon–target relationships were cross-referenced with differential expression results to establish regulatory networks.

**Ligand–Receptor Interaction Analysis (NicheNet)**

To predict intercellular signaling from macrophages to cardiomyocytes, we employed NicheNet (v1.0.0). As “sender” cells, we used DEGs from Cxcl9⁺ macrophages; as “receiver” cells, cardiomyocyte DEGs from bulk RNA-seq (Section 9). [7] The NicheNet predict_ligand_activities function was applied using default ligand–target model weights and background expression levels. Ligands were ranked by “ligand activity” scores, and target genes with regulation scores > 0.1 were retained. We constructed a ligand–target matrix focusing on the OSM–OSMR axis and visualized predicted links with chord diagrams. The expression of OSM in Cxcl9⁺ macrophages and corresponding receptor genes in cardiomyocytes was validated by scRNA-seq and bulk RNA-seq expression matrices.

**Cardiomyocyte Isolation and Functional Assays**

**Cardiomyocyte Isolation**

For adult cardiomyocyte isolation (AMCMs), adult mice were euthanized, and hearts were rapidly excised and mounted on a Langendorff perfusion apparatus via the aorta with retrograde flow (37 °C).[8] The heart was perfused first with calcium-free Tyrode’s buffer to flush blood, followed by digestion solution containing collagenase II (0.5 mg/mL) and protease (protease XIV) for ~10–15 min until the ventricles appeared soft and pale. After digestion, ventricles were minced, gently triturated, and filtered through a 100 µm mesh. Cells were collected and subjected to gradual calcium reintroduction (0.1, 0.2, 0.5, 1.0 mM CaCl₂) in stepwise increments to restore physiological calcium tolerance, as described in standard adult cardiomyocyte protocols to preserve viability and contractility. Viable, rod-shaped cardiomyocytes were enriched by gravity sedimentation and resuspended in plating medium (M199 supplemented with 2% bovine serum albumin and 5 mM creatine). Only cells exhibiting clear sarcomeric striations and quiescent morphology were used for downstream assays.[9]

For neonatal cardiomyocyte isolation (NMCMs), neonatal (P1–P3) mouse hearts were excised, atria removed, and ventricles minced into small fragments.[10] Tissue was digested in repeated cycles of 0.1% trypsin / collagenase II (Worthington) in Hank’s Balanced Salt Solution (HBSS) at 37 °C, with gentle agitation. After each digestion, cell suspensions were collected, filtered through 70 µm mesh, and pooled. Cells were preplated for 1 h in uncoated dishes to allow fibroblast adherence; the supernatant enriched in cardiomyocytes was collected and seeded on gelatin-coated plates in DMEM + 10% FBS + penicillin/streptomycin.

**Bulk RNA Sequencing of Cardiomyocytes**

Total RNA was extracted from freshly isolated AMCMs using TRIzol reagent (Invitrogen), followed by cleanup using RNeasy Mini Kit (Qiagen, Cat. No. 74106). RNA integrity was evaluated by Agilent 2100 Bioanalyzer (RIN > 8). Libraries were constructed using a standard poly(A) enrichment mRNA-seq kit (e.g., NEBNext Ultra II RNA Library Prep Kit). Sequencing was performed on an Illumina NovaSeq 6000 platform, generating 150-bp paired-end reads at a depth of ~30–50 million reads per sample. Raw reads were aligned to the mm10 mouse reference genome using STAR (v2.7), and gene-level counts quantified using featureCounts (Subread). Differential expression analysis was performed with DESeq2 (v1.36.0) using the moderated estimation of fold change and dispersion model. Genes with adjusted P < 0.05 and |log₂ fold change| ≥ 1 were considered significant. Functional enrichment (GO, KEGG) and GSEA (MSigDB hallmark gene sets) were applied to assess mitochondrial, contractile, and inflammatory gene programs.

**Conditioned Medium Co-culture Experiments**

Bone marrow–derived macrophages (BMDMs) were isolated from femurs/tibias of donor mice and cultured in RPMI 1640 + 10% FBS + M-CSF (20 ng/mL) for 7 days to mature.[11] For overexpression of Cebpb, BMDMs were transduced with adenoviral vectors encoding Cebpb (Ad-Cebpb) or control adenovirus (Ad-Null) at defined multiplicity of infection (MOI), incubated for 24–48 h. The culture medium was then replaced with low-serum (1% FBS) RPMI for 12 h, and the conditioned medium (CM) was collected, centrifuged to remove debris, and stored at 4 °C.

NMCMs or AMCMs were plated in multiwell plates and treated with 50% CM + 50% fresh medium, in the presence or absence of OSM-neutralizing antibody (2 µg/mL, R&D Systems) or isotype control, for 24 h. After treatment, cardiomyocytes were harvested for qPCR, ELISA, and Seahorse extracellular flux analysis according to manufacturer protocols (Seahorse XF96).

**Seahorse Metabolic Flux Analysis**

Mitochondrial respiration was assessed using the Seahorse XFe96 Extracellular Flux Analyzer (Agilent Technologies) with the Seahorse XF Cell Mito Stress Test Kit (Agilent, Cat# 103015-100) following the manufacturer’s instructions. Neonatal mouse cardiomyocytes (NMCMs) and bone-marrow-derived macrophages (BMDMs) were seeded in XF96 microplates (Agilent, Cat# 101085-004) precoated with 0.1 % gelatin (Sigma-Aldrich, Cat# G1890) at densities of 2 × 10⁴ cells/well (NMCMs) or 4 × 10^4^ cells/well (BMDMs). Before analysis, culture medium was replaced with Seahorse XF assay medium (Agilent, Cat# 103334-100) supplemented with 10 mM glucose (Agilent, Cat# 103577-100), 1 mM pyruvate (Agilent, Cat# 103578-100), and 2 mM L-glutamine (Agilent, Cat# 103579-100), pH 7.4, and incubated for 1 h at 37 °C in a non-CO_2_ incubator. Oxygen consumption rate (OCR) and extracellular acidification rate (ECAR) were measured at baseline and after sequential injections of oligomycin (1.5 μM), FCCP (1.0 μM), and rotenone/antimycin A (0.5 μM each) (Agilent, Cat# 103492-100, 103680-100, 103493-100, and 103694-100, respectively). Data were acquired and analyzed using Wave software (Agilent). Mitochondrial parameters—including basal respiration, ATP-linked respiration, maximal respiration, and spare respiratory capacity—were calculated according to manufacturer formulas. Each condition was tested in 3–5 replicate wells per plate, with results pooled from at least three independent experiments. OCR values were normalized to total protein content per well quantified using the BCA Protein Assay Kit (Abclonal, Cat. No. BRK0011).

**single-cell cardiomyocytes contraction assays**

Single ventricular cardiomyocytes were freshly isolated from mouse hearts by retrograde Langendorff perfusion as above described. Cell contractility was recorded using a video-based edge detection system (IonOptix MyoCam-S) under field stimulation. Myocytes were paced at 1 Hz with 4 ms square-wave pulses (20 V) using a pair of platinum electrodes connected to a MyoPacer stimulator (IonOptix). Sarcomere shortening was continuously monitored in unloaded conditions, and fractional shortening (% cell length change), contraction velocity (−dL/dt), and relaxation velocity (+dL/dt) were analyzed using IonWizard software (IonOptix). Only quiescent myocytes with stable baseline contraction and without spontaneous arrhythmic activity were included in the analysis. For each heart, at least 15–20 intact cardiomyocytes were examined, and data were averaged to represent one biological replicate.

**Co-immunoprecipitation (Co-IP) and Protein Interaction Analysis**

Macrophages (F4/80⁺ sorted from cardiac tissue) were lysed in ice-cold IP lysis buffer (50 mM Tris-HCl pH 7.5, 150 mM NaCl, 1% NP-40, 1 mM EDTA) supplemented with protease and phosphatase inhibitor cocktails (Abclonal, Cat. No. RM02997). Lysates were cleared by centrifugation (14,000 × g, 15 min, 4 °C), and protein concentrations were measured by BCA assay. For immunoprecipitation, equal amounts (500–1,000 µg) of total protein were incubated with 2–5 µg of primary anti-Raptor (CST, Cat. No. 2280) overnight at 4 °C with gentle rotation. Subsequently, 30 µL of Protein A/G magnetic beads (Thermo Fisher, Cat. No. 10001D) were added and incubated for 2 h at 4 °C. Beads were washed 4 times with lysis buffer, then boiled in SDS loading buffer to elute bound proteins. Eluates, along with input controls (5–10% of lysate), were resolved by SDS-PAGE and immunoblotted with antibodies against C/EBPβ (CST, Cat. No. 3087), p-C/EBPβ (CST, Cat. No. 3084) and Raptor (Abclonal, Cat. No. A8992) as required. Detection was carried out via HRP-conjugated secondary antibodies and ECL chemiluminescence.

**Statistical Analysis**

Statistical analyses were performed using GraphPad Prism (v10) and R (v4.3). Normality was assessed with the Shapiro–Wilk test. Continuous data are presented as mean ± SEM (normally distributed) or median [IQR] (non-normal). Two-group comparisons used two-tailed Student’s *t* test (parametric) or Mann–Whitney *U* test (non-parametric). Comparisons among ≥3 groups used one-way or two-way ANOVA with Tukey’s or Bonferroni post-hoc tests, as appropriate. Categorical variables were compared with χ^2^ or Fisher’s exact tests. Correlations used Spearman’s ρ. For Seahorse assays and repeated measurements, between-group effects were evaluated by two-way ANOVA with interaction terms and appropriate multiple-comparison correction. For single-cell analyses, quality control, normalization, dimensionality reduction, clustering, and differential expression followed standard pipelines, with gene-level statistics obtained by Wilcoxon rank-sum tests and Benjamini–Hochberg false-discovery rate (FDR) correction. Gene-set scoring used AddModuleScore/AUCell, and pathway enrichment employed clusterProfiler/fgsea with FDR control. Pseudotime trajectories were inferred with Monocle 3; regulon activity was computed via SCENIC/AUCell; ligand–receptor inference used NicheNet. Subset enrichment/odds ratios were estimated from cell-type proportions using logistic models with cluster-robust standard errors; 95% confidence intervals are reported where applicable. Unless otherwise stated, tests were two-sided, and *P* < 0.05 (or FDR q < 0.05 for multi-gene analyses) was considered statistically significant.

**Supplementary figures**

**Figure S1. Single-cell transcriptomic profiling reveals rapamycin-induced remodeling of cardiac immune and stromal populations in EAM.**


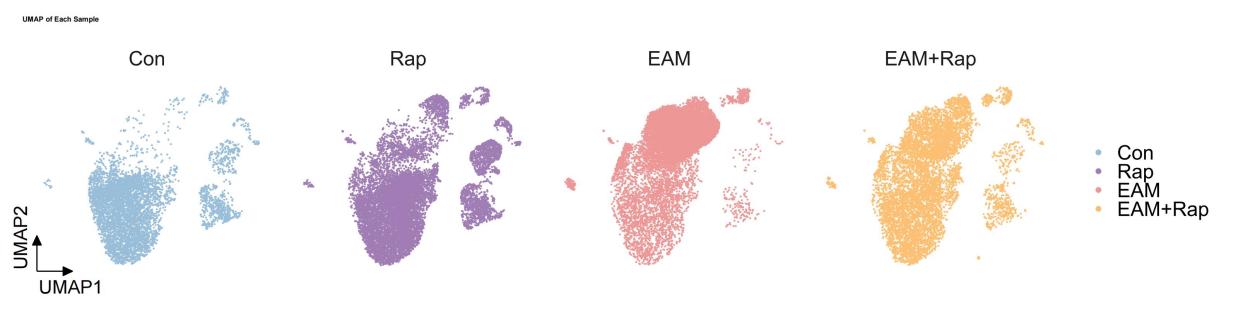


**A**

**B**


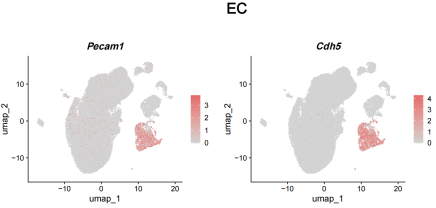

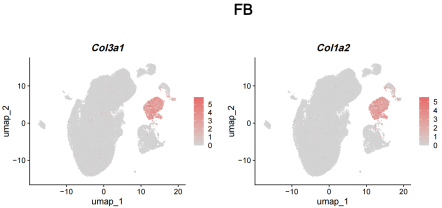

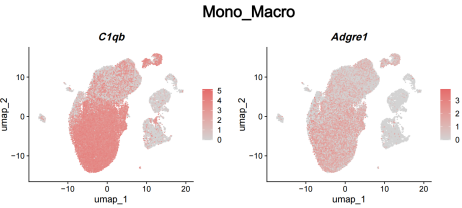

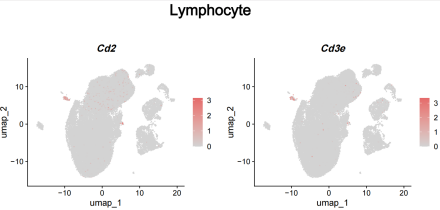

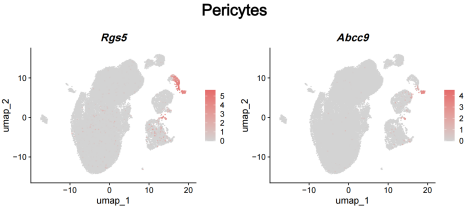

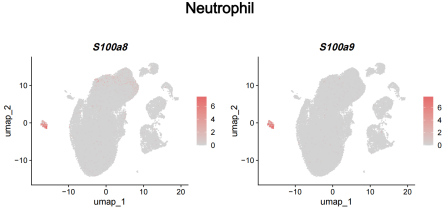


**D**


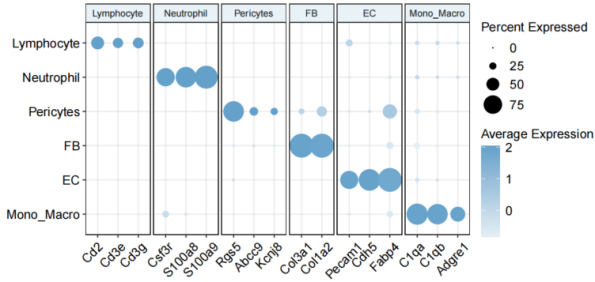


**C**


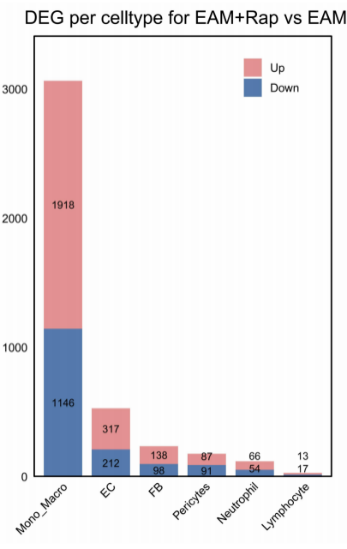


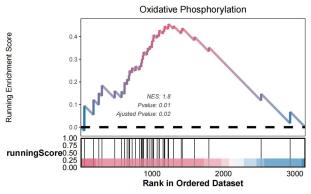

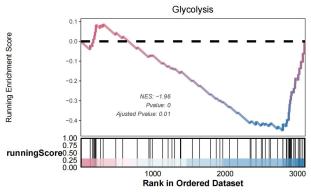

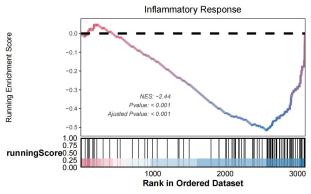

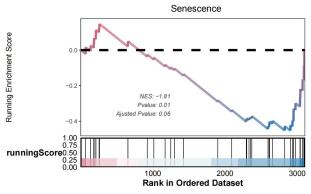


**E**

**F**


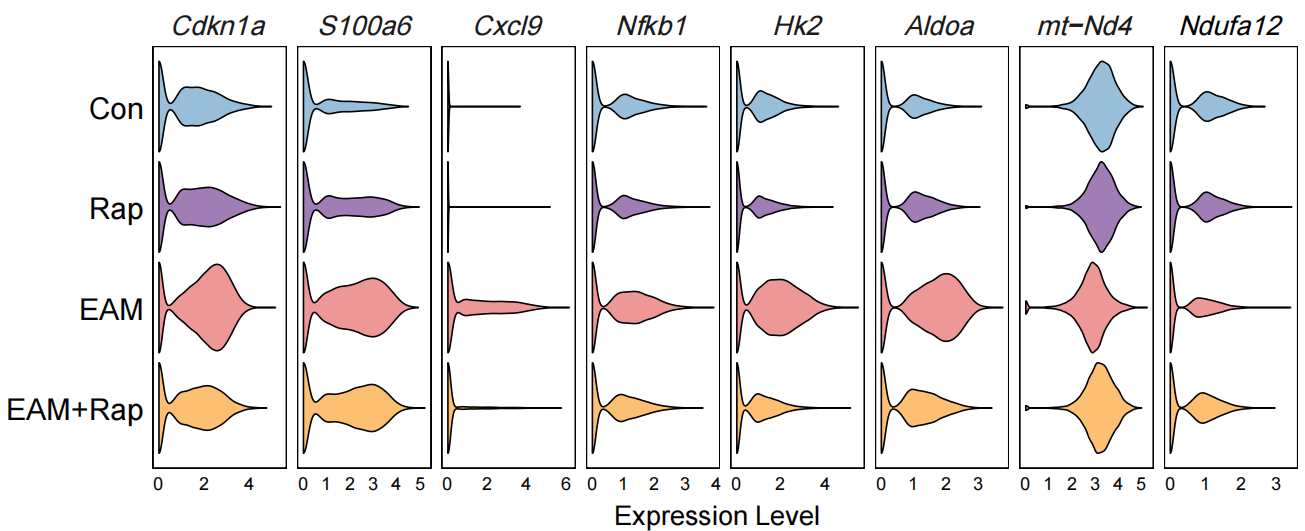


**G**


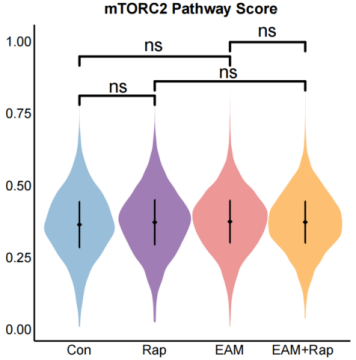


(**A**) UMAP visualization of scRNA-seq data derived from cardiac CD45⁺ immune cells isolated from control (Con), rapamycin-treated (Rap), experimental autoimmune myocarditis (EAM), and combined EAM + Rap mice, showing global cellular distribution patterns. (**B**) Feature plots of representative canonical marker genes used for identification of major cardiac cell populations, including myeloid cells (*C1qb*, *Adgre1*), neutrophils (*S100a8*, *S100a9*), lymphocytes (*Cd3e*, *Cd79a*), pericytes (*Rgs5*, *Abcc9*), endothelial cells (*Pecam1*, *Cdh5*), and fibroblasts (*Col1a1*, *Col1a2*). (**C**) Dot plot showing the relative expression levels and proportion of cells expressing canonical marker genes across identified cell clusters. (**D**) Quantification of DEGs per cell type in EAM + Rap versus EAM, demonstrating the most extensive rapamycin-responsive transcriptional reprogramming within the monocyte–macrophage (Mono_Macro) compartment. (**E**) GSEA plots illustrating enrichment of representative biological pathways in EAM versus EAM + Rap macrophages, including senescence, glycolysis, inflammatory response, and OXPHOS. (**F**) Violin plots depicting normalized expression levels of representative genes associated with cellular senescence (*Cdkn1a*), inflammation (*S100a6*, *Cxcl9*, *Nfkb1*), glycolytic metabolism (*Hk2*, *Aldoa*), and mitochondrial oxidative phosphorylation (*mt-Nd4*, *Ndufa12*) across experimental groups. (**G**) Violin plots depict mTORC2 pathway scores in cardiac macrophages from Control + Vehicle (Con), Control + Rapamycin (Rap), EAM + Vehicle (EAM), and EAM + Rapamycin (EAM+Rap) groups. Scores were calculated using gene set–based pathway scoring for an mTORC2-related signature. Center lines indicate median values with interquartile ranges. Statistical comparisons were performed by one-way ANOVA followed by Tukey’s multiple-comparison test; ns, not significant.

**Abbreviations**: EAM, experimental autoimmune myocarditis; Rap, rapamycin; scRNA-seq, single-cell RNA sequencing; UMAP, uniform manifold approximation and projection; DEGs, differentially expressed genes; GSEA, gene set enrichment analysis; OXPHOS, oxidative phosphorylation; Mono_Macro, monocyte–macrophage.

**Figure S2. Rapamycin selectively reprograms the transcriptional and metabolic signatures of Cxcl9⁺ macrophages in EAM.**


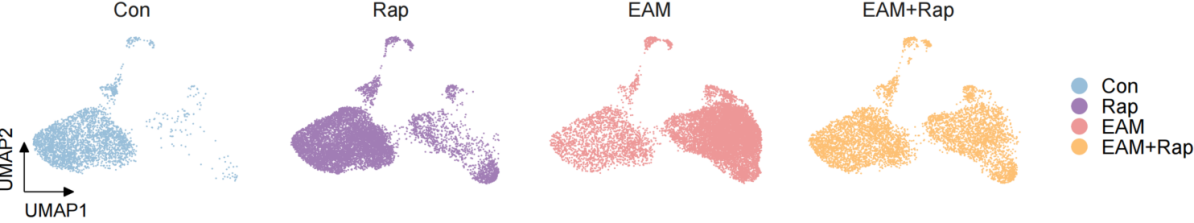


**A**


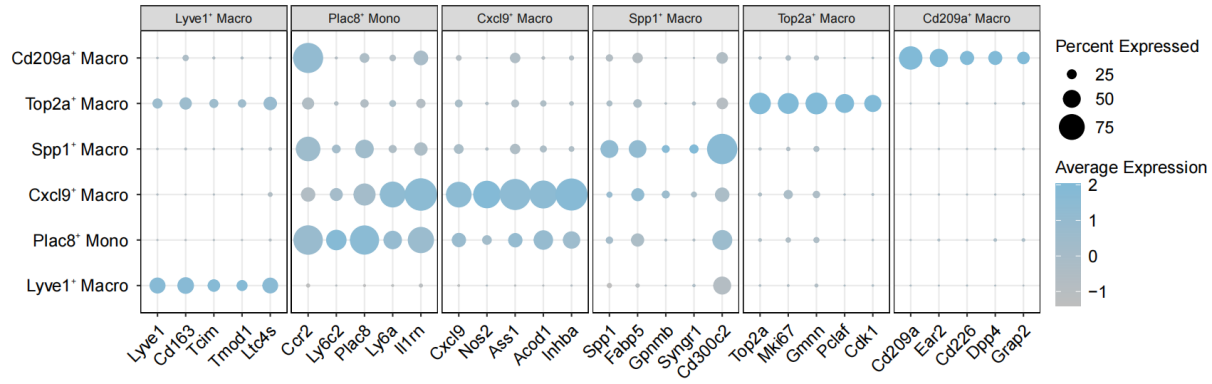


**B**


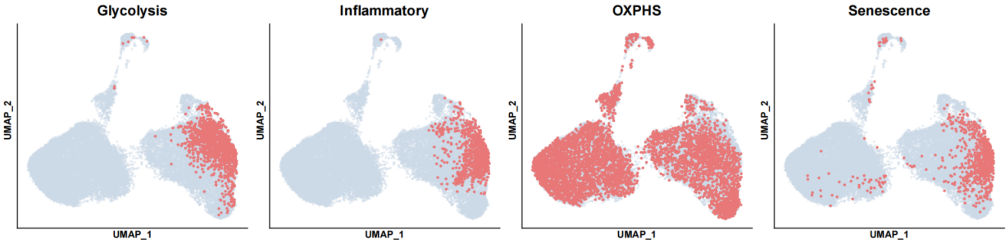


**D**


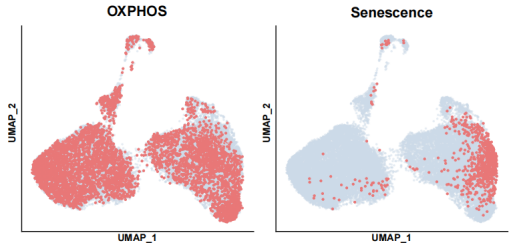

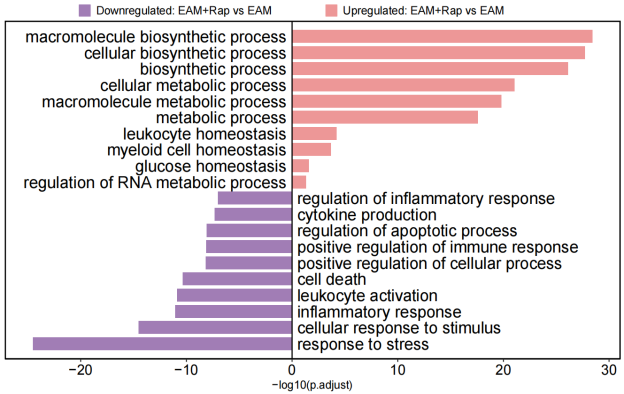


**C**


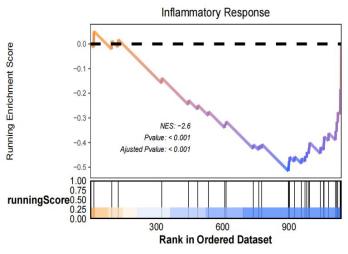

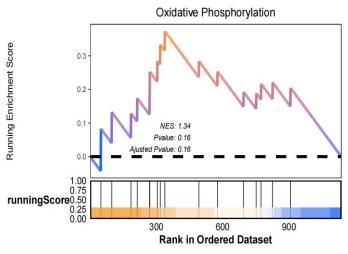

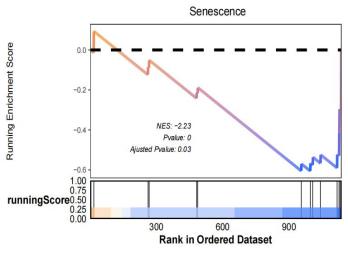

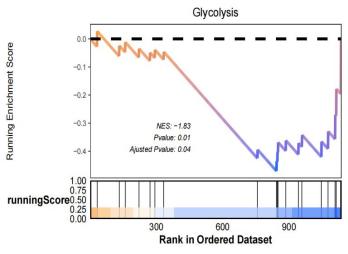


**E**

**F**


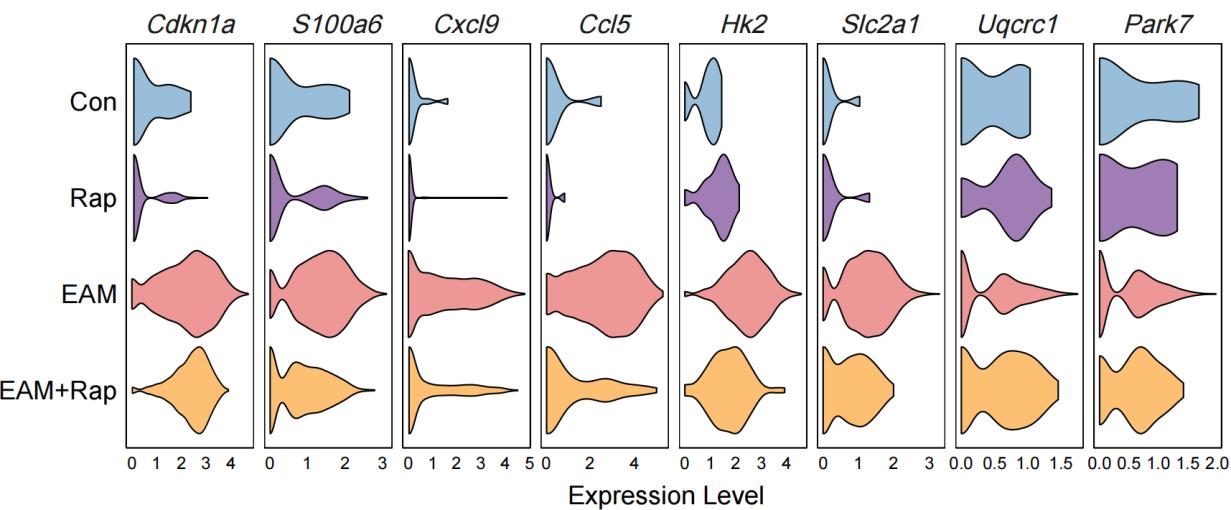


(**A**) UMAP visualization of reclustered cardiac monocyte–macrophage populations from Control (Con), Rapamycin (Rap), Experimental Autoimmune Myocarditis (EAM), and EAM + Rap groups. (**B**) Dot plot showing canonical marker genes used for identification of six macrophage subsets, including Lyve1⁺ macrophages, Plac8⁺ monocytes, Cxcl9⁺ inflammatory macrophages, Spp1⁺ remodeling macrophages, Top2a⁺ proliferative macrophages, and Cd209a⁺ antigen-presenting macrophages. (**C**) GO enrichment analysis of DEGs in Cxcl9⁺ macrophages comparing EAM and EAM + Rap groups, showing downregulation of inflammatory and stress-response pathways and upregulation of biosynthetic and metabolic processes. (**D**) UMAP feature plots of pathway activity scores illustrating distribution of glycolysis, OXPHOS, inflammatory, and senescence signatures across macrophage subsets. (**E**) GSEA plots showing enrichment of glycolysis, senescence, oxidative phosphorylation, and inflammatory response pathways in Cxcl9⁺ macrophages between EAM and EAM + Rap groups. (**F**) Violin plots showing normalized expression levels of representative genes related to senescence (*Cdkn1a, S100a6*), inflammation (*Cxcl9, Ccl5*), glycolysis (*Hk2, Slc2a1*), and mitochondrial oxidative phosphorylation (*Uqcrc1, Park7*) across experimental groups.

**Abbreviations:** EAM, experimental autoimmune myocarditis; Rap, rapamycin; UMAP, uniform manifold approximation and projection; scRNA-seq, single-cell RNA sequencing; GO, Gene Ontology; DEG, differentially expressed gene; GSEA, gene set enrichment analysis; OXPHOS, oxidative phosphorylation; CXCL9, C-X-C motif chemokine ligand 9.

**Figure S3. Pseudotime and transcriptional analyses reveal rapamycin-mediated remodeling of macrophage differentiation and C/EBPβ-dependent inflammatory programming in EAM.**


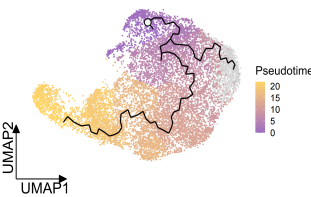


**C**


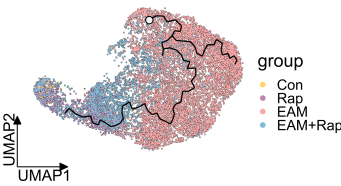


**A**


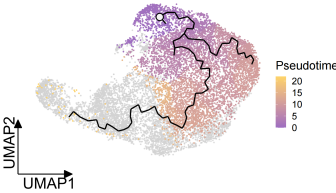


**B**

**D**


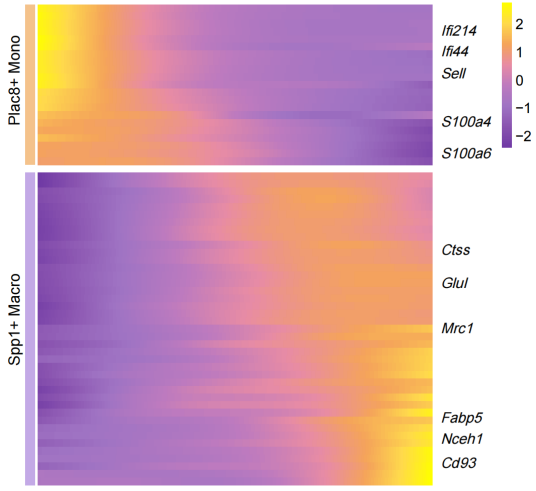

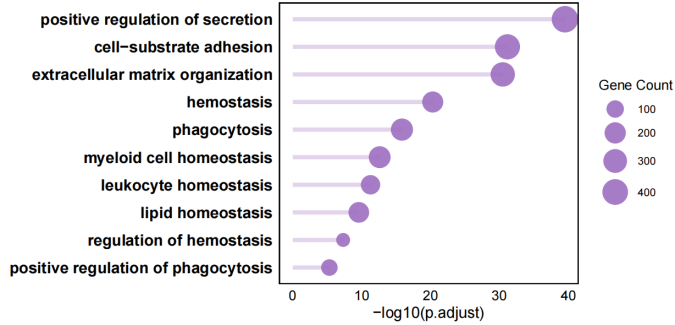


**E**


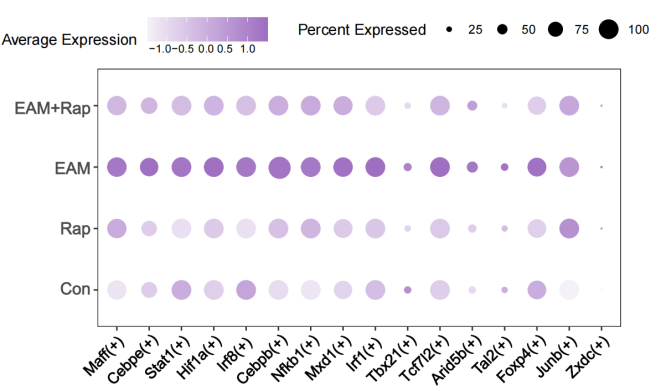


**F**


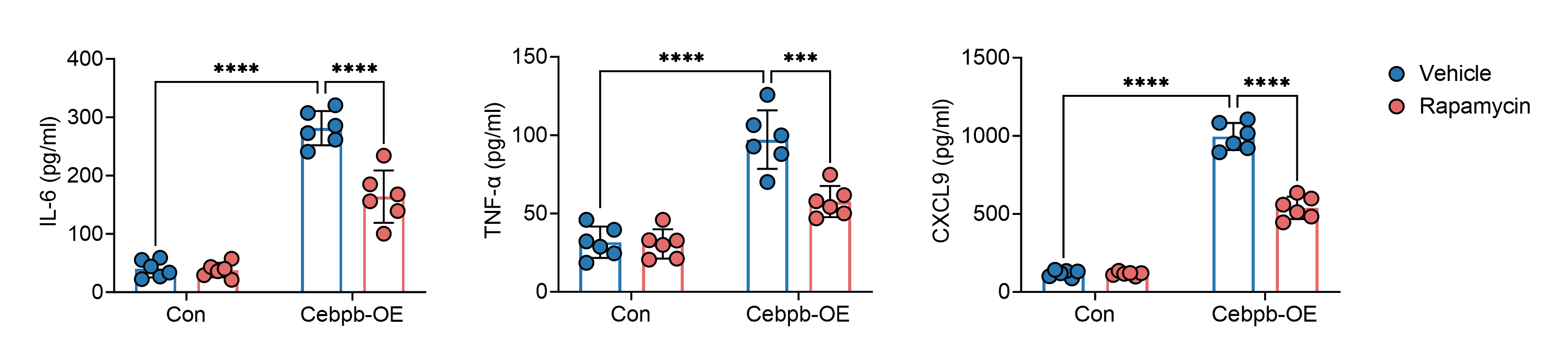


**G**

(**A**) UMAP visualization of monocyte–macrophage subsets colored by experimental group [Control (Con), Rapamycin (Rap), EAM, and EAM + Rap]. (**B**–**C**) Monocle3-inferred pseudotime trajectory of macrophage differentiation showing two major branches derived from Plac8⁺ monocytes: one progressing toward Cxcl9⁺ inflammatory macrophages and the other toward Spp1⁺ reparative macrophages. (**D**) Heatmaps of dynamically regulated genes along the Plac8⁺ and Spp1⁺ branches, highlighting representative lineage-associated gene programs. (**E**) GO enrichment analysis of pseudotime-dependent genes showing enrichment for biological processes related to secretion, extracellular matrix organization, homeostasis, and phagocytosis. (**F**) Dot plot showing relative expression levels and proportion of cells expressing representative transcriptional regulators (e.g., *Irf1, Hif1a, Cebpb, Nfkb1*) across experimental groups. (**G**) ELISA quantification of IL-6, TNF-α, and CXCL9 levels in culture supernatants from BMDMs transduced with control or Cebpb-overexpressing (*Cebpb*-OE) adenovirus, treated with vehicle or rapamycin (n = 6 per group).

**Abbreviations:** EAM, experimental autoimmune myocarditis; Rap, rapamycin; UMAP, uniform manifold approximation and projection; GO, Gene Ontology; IL, interleukin; TNF, tumor necrosis factor; CXCL9, C-X-C motif chemokine ligand 9; BMDM, bone marrow–derived macrophage; OE, overexpression;

**Figure S4. Osmr expression in isolated cardiomyocytes from control and EAM mice treated with vehicle or rapamycin.**


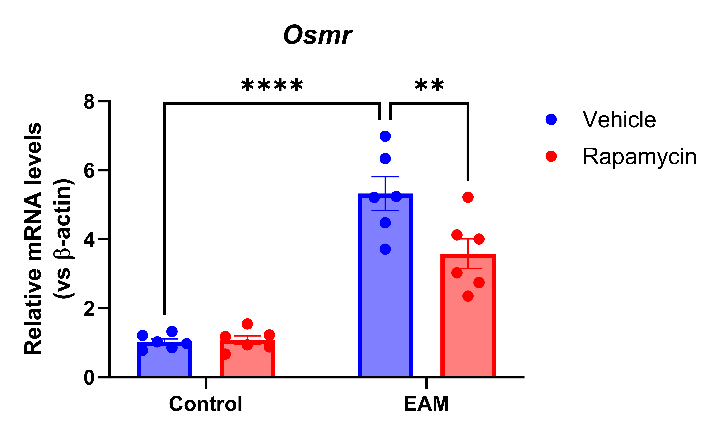


Relative *Osmr* mRNA levels (normalized to β-actin) were measured by RT-qPCR in cardiomyocytes isolated from Langendorff-perfused hearts of Control + Vehicle, Control + Rapamycin, EAM + Vehicle, and EAM + Rapamycin groups (n = 5 per group). Data are presented as mean ± SEM. ***P* < 0.01, *****P* < 0.0001 by two-way ANOVA followed by Tukey’s multiple-comparison test.

Abbreviations: EAM, experimental autoimmune myocarditis; RT-qPCR, reverse transcription quantitative PCR; SEM, standard error of the mean; ANOVA, analysis of variance.

**Supplemental References**

[1] X. Hua, G. Hu, Q. Hu, Y. Chang, Y. Hu, L. Gao, X. Chen, P.C. Yang, Y. Zhang, M. Li, J. Song, Single-Cell RNA Sequencing to Dissect the Immunological Network of Autoimmune Myocarditis, Circulation. 142 (2020) 384-400. <https://doi.org/10.1161/CIRCULATIONAHA.119.043545>.

[2] T.M. Marin, K. Keith, B. Davies, D.A. Conner, P. Guha, D. Kalaitzidis, X. Wu, J. Lauriol, B. Wang, M. Bauer, R. Bronson, K.G. Franchini, B.G. Neel, M.I. Kontaridis, Rapamycin reverses hypertrophic cardiomyopathy in a mouse model of LEOPARD syndrome-associated PTPN11 mutation, J Clin Invest. 121 (2011) 1026-1043. <https://doi.org/10.1172/JCI44972>.

[3] B. Carames, A. Hasegawa, N. Taniguchi, S. Miyaki, F.J. Blanco, M. Lotz, Autophagy activation by rapamycin reduces severity of experimental osteoarthritis, Ann Rheum Dis. 71 (2012) 575-581. <https://doi.org/10.1136/annrheumdis-2011-200557>.

[4] X. Zhang, S. Yuan, H. Li, J. Zhan, F. Wang, J. Fan, X. Nie, Y. Wang, Z. Wen, Y. Chen, C. Chen, D.W. Wang, The double face of miR-320: cardiomyocytes-derived miR-320 deteriorated while fibroblasts-derived miR-320 protected against heart failure induced by transverse aortic constriction, Signal Transduct Target Ther. 6 (2021) 69. <https://doi.org/10.1038/s41392-020-00445-8>.

[5] H. Li, M. Zhang, Q. Zhao, W. Zhao, Y. Zhuang, J. Wang, W. Hang, Z. Wen, L. Wang, C. Chen, D.W. Wang, Self-recruited neutrophils trigger over-activated innate immune response and phenotypic change of cardiomyocytes in fulminant viral myocarditis, Cell Discov. 9 (2023) 103. <https://doi.org/10.1038/s41421-023-00593-5>.

[6] J. Wen, H. Li, Y. Zhou, H. Du, G. Hu, Z. Wen, D. Tang, Y. Wang, X. Cui, Z. Zhou, D.W. Wang, C. Chen, Immunoglobin attenuates fulminant myocarditis by inhibiting overactivated innate immune response, Br J Pharmacol. 182 (2025) 4984-5003. <https://doi.org/10.1111/bph.17372>.

[7] C. Panico, A. Felicetta, P. Kunderfranco, M. Cremonesi, N. Salvarani, P. Carullo, F. Colombo, A. Idini, M. Passaretti, R. Doro, M. Rubino, A. Villaschi, G. Da Rin, C. Peano, M. Kallikourdis, C.M. Greco, G. Condorelli, Single-Cell RNA Sequencing Reveals Metabolic Stress-Dependent Activation of Cardiac Macrophages in a Model of Dyslipidemia-Induced Diastolic Dysfunction, Circulation. 150 (2024) 1517-1532. <https://doi.org/10.1161/CIRCULATIONAHA.122.062984>.

[8] J. Fan, H. Li, R. Xie, X. Zhang, X. Nie, X. Shi, J. Zhan, Z. Yin, Y. Zhao, B. Dai, S. Yuan, Z. Wen, C. Chen, D.W. Wang, LncRNA ZNF593-AS Alleviates Contractile Dysfunction in Dilated Cardiomyopathy, Circ Res. 128 (2021) 1708-1723. <https://doi.org/10.1161/CIRCRESAHA.120.318437>.

[9] H. Du, Y. Zhao, J. Wen, B. Dai, G. Hu, Y. Zhou, Z. Yin, N. Ding, H. Li, J. Fan, X. Nie, F. Wang, Q. Liu, Z. Wen, G. Xu, D.W. Wang, C. Chen, LncRNA DCRT Protects Against Dilated Cardiomyopathy by Preventing NDUFS2 Alternative Splicing by Binding to PTBP1, Circulation. 150 (2024) 1030-1049. <https://doi.org/10.1161/CIRCULATIONAHA.123.067861>.

[10] J. Zhan, K. Jin, R. Xie, J. Fan, Y. Tang, C. Chen, H. Li, D.W. Wang, AGO2 Protects Against Diabetic Cardiomyopathy by Activating Mitochondrial Gene Translation, Circulation. 149 (2024) 1102-1120. <https://doi.org/10.1161/CIRCULATIONAHA.123.065546>.

[11] A.M. Luu, K.M. Shepardson, A. Rynda-Apple, A Comprehensive Protocol for the Collection, Differentiation, Cryopreservation, and Resuscitation of Primary Murine Bone Marrow Derived Macrophages (BMDM), Immunol Invest. 53 (2024) 1001-1012. <https://doi.org/10.1080/08820139.2024.2382805>.
